# Brief Drug Interventions Delivered in General Medical Settings: a Systematic Review and Meta-analysis of Cannabis Use Outcomes

**DOI:** 10.1007/s11121-025-01826-7

**Published:** 2025-07-08

**Authors:** Lauren M. Berny, Lindsey M. Nichols, Maria L. Schweer-Collins, Emily E. Tanner-Smith

**Affiliations:** 1 Department of Counseling Psychology and Human Services, University of Oregon, Eugene, OR 97403 USA; 2 Prevention Science Institute, University of Oregon, Eugene, OR 97403 USA; 3 The Warren Alpert Medical School, Brown University, Providence, RI 02903 USA; 4 Department of Psychology, Iowa State University, Ames, IA 50011 USA; 5 Institute for Evidence-Based Educational Practice, HEDCO, University of Oregon, Eugene, OR 97403 USA

**Keywords:** Brief interventions, Cannabis, Healthcare, Meta-analysis

## Abstract

There is consistent evidence that brief interventions can be effective in preventing and reducing alcohol use, but support for their effects on illicit drug use is more limited. This meta-analysis expands prior research by testing whether brief drug interventions (BDIs) delivered in general medical settings reduce cannabis consumption and severity across post-intervention follow-up periods and explores potential heterogeneity in these effects. Findings from 17 randomized controlled trials were synthesized to compare short- and long-term cannabis use outcomes between intervention and control groups. Mixed effects meta-regression models were estimated to examine variability in effects across four intervention characteristics: booster session delivery, delivery setting, intervention target, and target population. Sensitivity tests were also conducted for both main effects and moderation analyses. There was no evidence that BDIs yielded significant short-term reductions in cannabis use (*OR* = 1.20, 95% CI [0.90, 1.62]), consumption level (*g* = 0.01, 95% CI [− 0.07, 0.09]), or severity (*g* = 0.13, 95% CI [− 0.07, 0.33]). Similarly, there was no evidence of effects on long-term cannabis use (*OR* = 1.19, 95% CI [0.73, 1.86]) or consumption level (*g* = 0.04, 95% CI [− 0.05, 0.12]). Although the primary analyses did not provide evidence of effect moderation, sensitivity tests revealed BDIs delivered in emergency departments evidenced small but significant reductions in long-term cannabis consumption levels. While these findings do not provide support for the overall effectiveness of BDIs on cannabis consumption or severity in general medical settings, they offer suggestive evidence that BDIs may perform more favorably when delivered in emergency departments. Thus, high-quality trials evaluating the effects of BDIs in emergency departments are needed. Given the importance of preventing adverse health outcomes and consequences of cannabis use, more research is needed to improve and evaluate BDI outcomes. Further, developing and testing alternative prevention and intervention approaches are essential to comprehensively address cannabis use.

**Supplementary Information:**

The online version contains supplementary material available at 10.1007/s11121-025-01826-7.

Cannabis use in the USA has changed dramatically as legal recreational and medical availability of the drug has expanded. Cannabis use has increased in the general population, with past-month use among those aged 12 and older increasing by 38% between 2015 and 2019 (Palamar et al., 2021). Cannabis use disorder is also now prevalent: an estimated 40.3% of those aged 12 and older who reported past-month cannabis use met diagnostic criteria for cannabis use disorder (Substance Abuse and Mental Health Services Administration [SAMHSA], 2023b). Adverse health effects of frequent cannabis use may include cardiovascular and respiratory complications, poor sleep quality, decision-making deficits, and depression and anxiety symptom severity (Fischer et al., 2022; Leadbeater et al., 2019; Lovell et al., 2020). Frequent cannabis use during adolescence, in particular, has been associated with poor health and psychosocial outcomes (Hall & Degenhardt, 2009), including reduced educational attainment, increased risk of cannabis dependence, other illicit drug use, criminal activity, internalizing disorder symptoms, and suicidality (Degenhardt et al., 2013; Fergusson et al., 2002; Silins et al., 2014). Despite these consequences, treatment rates for cannabis use disorder are low. Only 7.8% of US adults who met criteria for cannabis use disorder received cannabis-specific treatment in the past year (Wu et al., 2017), and the most common reasons for not receiving treatment include lack of perceived need, affordability, and accessibility (Kerridge et al., 2017). Given the prevalence and consequences of cannabis use, interventions that address these barriers to treatment may be critical for identifying and preventing frequent cannabis use.

One such model is the brief intervention, defined here as a counseling intervention delivered in four or fewer sessions that aims to increase awareness of substance use consequences and thereby reduce consumption. As a whole, brief interventions are heterogenous in their approach; other than their brevity, they can vary in structure, core components, and delivery methods. Some brief interventions simultaneously target co-occurring alcohol and drug use, whereas others may focus solely on a single substance (e.g., cannabis); such flexibility allows brief interventions to be administered to broader populations or tailored to specific groups (Mattoo et al., 2018; Roche & Freeman, 2004). Although brief interventions can be delivered in a variety of settings, embedding them into services in general medical settings—such as emergency departments, community health centers, primary care clinics, and student health centers—presents a potential opportunity to make substance use prevention and referral to treatment more accessible. Because substance use disorders are often overrepresented among individuals who receive clinical services in emergency departments and primary care clinics (Cherpitel & Ye, 2008; Pilowsky & Wu, 2012), these general medical settings present well-timed opportunities to address substance use among those who may not otherwise seek treatment. Nonetheless, clinicians in general medical settings who are well-positioned to screen for and discuss substance use with their patients often have minimal time and resources beyond treating their presenting conditions. Thus, brief interventions may be appealing given their short duration, limited training requirements, and possible cost-efficiency (Barbosa et al., 2017). The potential reach, adaptability, and feasibility of brief interventions delivered in general medical settings therefore make them a pragmatic approach to opportunistically address cannabis use.

Although reviews of brief alcohol interventions have shown beneficial if modest reductions in drinking (Kaner et al., 2018), support for brief drug interventions (BDIs) is more limited (Saitz, 2014). Recent meta-analyses of BDIs have revealed mixed results, showing evidence of null or modest beneficial effects on drug use (Halladay et al., 2019; Imtiaz et al., 2020; Sahker et al., 2022; Tanner-Smith et al., 2022). These mixed results may be due to variability in BDI effects across different study and intervention characteristics. One important study characteristic is the setting in which the intervention is delivered. For example, a recent meta-analysis found no evidence that brief interventions delivered in outpatient medical facilities reduced frequency of drug use, but effects significantly varied across delivery settings, with subgroup analyses revealing that those delivered in emergency departments yielded small positive reductions in drug use frequency (Sahker et al., 2022). BDIs may also have greater beneficial effects in certain populations and/or when specifically targeting specific substances. For instance, another recent meta-analysis found that cannabis-targeted BDIs for adolescents and young adults were associated with small reductions in short-term cannabis use disorder symptoms and higher odds of short-term abstinence (Halladay et al., 2019). However, the same meta-analysis found no evidence of reductions in cannabis use frequency or consequences of cannabis use, suggesting results may vary across outcomes, domains, or measures. Differences in the therapeutic components of interventions are also important to consider, such as whether interventions include booster sessions to help sustain intervention effects. Although booster sessions are often employed with brief interventions, evidence about their impact is mixed. For instance, when evaluating the effects of brief interventions delivered in general medical settings, Tanner-Smith et al. (2022) found that delivery of a booster in brief alcohol interventions was associated with significant reductions in alcohol use, whereas BDIs delivered without a booster had larger beneficial reductions in mixed substance use (i.e., alcohol and other drug use) compared to BDIs with a booster. Thus, given the variation in BDI effects, attempts to synthesize the literature on BDIs should attend carefully to such potential heterogeneity in effects.

Given the potential consequences of cannabis use and low receipt of treatment for cannabis-related problems, the primary aim of this meta-analysis is to evaluate whether BDIs delivered opportunistically in general medical settings result in beneficial reductions in the consumption and severity of cannabis use. As a secondary aim, this meta-analysis examines the variability in effects across patient populations, settings, intervention targets, and booster session delivery. The current meta-analysis builds upon and makes important contributions to the existing literature. Two recent meta-analyses examined the effects of brief interventions delivered specifically in healthcare settings on cannabis outcomes. First was a synthesis of findings from six studies examining the effects of BDIs delivered in healthcare settings with patients who reported recent cannabis use, which reported no evidence of effects on frequency nor severity of cannabis use (Imtiaz et al., 2020). Second was a review synthesizing findings from 111 studies of brief interventions delivered in general medical settings, which reported a subgroup analysis among 13 studies indicating small beneficial effects of BDIs on cannabis use that attenuated to non-significance after adjusting for multiple comparisons (Tanner-Smith et al., 2022). Both of these reviews suggested that BDIs delivered in general medical settings may have modest to minimal effects on cannabis consumption outcomes. However, neither of these reviews conducted in-depth analyses to examine whether BDI effects on cannabis use might vary across study, population, or intervention characteristics for different cannabis use outcomes. Given the likely variability in the ways BDIs are implemented in different general medical settings, it is essential to explore such potential heterogeneity before concluding they have consistently small or null effects on cannabis use. The present meta-analysis thus expands the current evidence base using data from Tanner-Smith et al. (2022) by examining multiple cannabis use outcomes across different post-intervention follow-up periods as well as estimating meta-regression models to further explore heterogeneity in BDI effects.

## Methods

Methods and findings are reported following the PRISMA 2020 reporting guidelines (Page et al., 2021). Protocols for both the parent systematic review and meta-analysis as well as this secondary analysis were pre-registered and posted on Open Science Framework. There were two post hoc deviations from the analysis protocol that were exploratory in nature, as described in greater detail below: (1) additional sensitivity tests to assess the impact of effect size selection based on follow-up time and intervention modality; and (2) applying effect size selection sensitivity tests to moderation analyses.

### Eligibility Criteria and Search Strategy

**Fig. 1:**
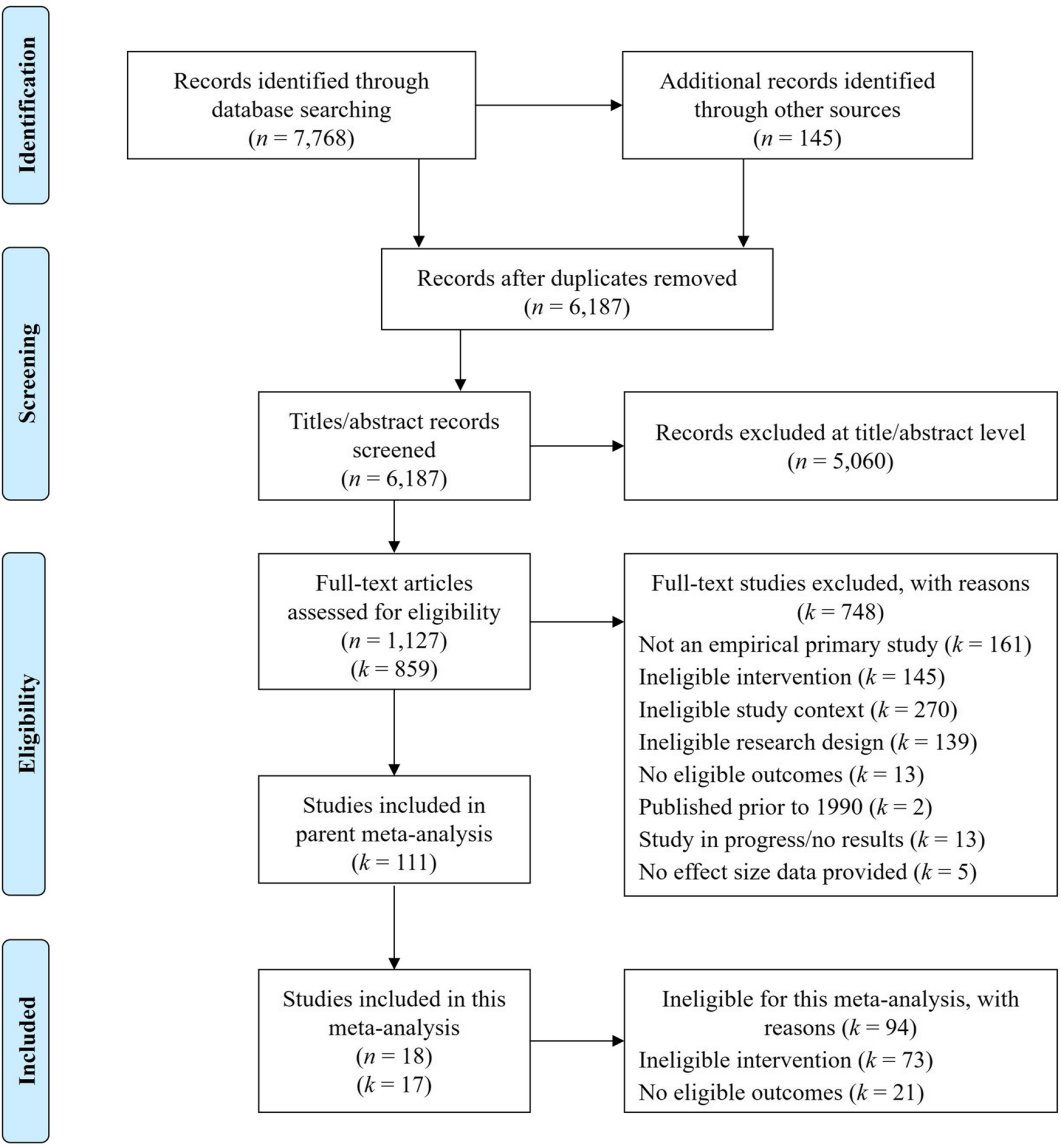
PRISMA flow diagram displaying numbers of reports and studies included in review

This meta-analysis analyzes a subset of studies from a larger parent meta-analysis evaluating the effectiveness of brief substance use interventions delivered in general medical settings (Tanner-Smith et al., 2022). The parent meta-analysis included brief interventions targeting alcohol use, other drug use, or mixed substance use (i.e., both alcohol and other drug use). To be eligible for inclusion in the parent meta-analysis, studies had to evaluate brief interventions delivered in four or fewer sessions to patients recruited from general, non-specialized medical service settings. Eligible studies were required to use a randomized controlled trial design to compare a brief intervention with a less active comparison condition (e.g., no treatment and treatment as usual) and report at least one post-intervention measure of substance use or substance use–related consequences. To be eligible for inclusion in the present analytic sample, studies were required to evaluate the effects of a BDI—defined here as explicitly targeting drug use either singularly or in conjunction with alcohol use—and report post-intervention measures of cannabis consumption or severity. Thus, studies were ineligible if they evaluated interventions that only targeted alcohol during the intervention (Bernstein et al., 2010; Cancilliere et al., 2018). See Fig. 1 for a study selection flow diagram.

The parent meta-analysis used a comprehensive search strategy to identify relevant published and unpublished studies, which yielded the studies considered for inclusion in the present meta-analysis. The following databases (hosts) were searched from 1990 through March 31, 2020: PubMed; Nursing/Academic Edition (EBSCO); ERIC, Applied Social Sciences Index and Abstracts, Dissertations and Theses Global, Social Services Abstract (ProQuest); PsycINFO (PsycNET); Cochrane Central Register of Controlled Trials; World Health Organization (WHO) International Clinical Trials Registry; and National Institutes of Health (NIH) RePORTER. Reference lists of all screened and eligible studies and those in prior reviews were examined. Hand searches were also performed of the 1990 to 2020 table of contents in *Addiction*, *Addictive Behaviors*, *Campbell Systematic Reviews*, and *Journal of Studies on Alcohol and Drugs*.

### Study Selection and Data Extraction

A rigorous three-stage study selection and data extraction process was conducted by trained research assistants as part of the parent meta-analysis’ protocol. First, two reviewers independently screened titles and abstracts to eliminate studies that did not meet the search criteria. Any reports considered potentially relevant for inclusion by at least one reviewer proceeded to the next stage, and the Principal Investigator double-screened any reports deemed ineligible. At the second stage, two reviewers independently reviewed the full texts to determine final eligibility, and any disagreements were resolved through discussion with the Principal Investigator. At the third stage of data extraction for eligible studies, two reviewers independently coded and extracted data, and any disagreements were resolved through discussion with the Principal Investigator. This data extraction process followed a pre-registered, standardized coding protocol (see Tanner-Smith et al., 2022 for additional details). The Cochrane Collaboration’s risk of bias tool (ROB 1.0) for randomized controlled trials was used to assess risk of bias in the included studies (Higgins et al., 2011).

#### Outcomes

Outcomes were assessed separately for short-term (0–5 month post-intervention) and long-term follow-up (6–12 months post-intervention). To comprehensively examine BDI effects on cannabis use, we examined three outcomes: (1) consumption levels (continuous outcome), defined as the number of days cannabis was used or the quantity of cannabis used; (2) general use (binary outcome), defined as any cannabis use; and (3) severity of use (continuous outcome), defined as the summed total of seven cannabis-specific items on the Alcohol, Smoking and Substance Involvement Screening Test (ASSIST; WHO ASSIST Working Group, 2002). Supplemental Material 1 details the outcome measures synthesized in this meta-analysis.

#### Moderators

We examined the following potential effect size moderators: (1) delivery of a booster session (yes vs. no); (2) primary setting (emergency department, community health centers, student health centers, hospital-based primary care, or multiple settings); (3) BDI target (BDIs that targeted cannabis vs. BDIs that targeted mixed drugs or drugs other than cannabis); and (4) sample population (adolescents only, adolescents and young adults, mixed/adults only, and university students). University students were assessed as a separate subpopulation because there is a growing body of literature evaluating BDIs specifically designed for university students and implemented in that setting; mixed and adult-only samples were combined for analysis due to their similar average ages. For BDI target, the first author (LMB) reviewed and coded the eligible studies to determine whether the BDIs specifically targeted cannabis use or other drug/mixed substance use. Following that review, the second author (LMN) reviewed the coding for consistency. All other moderators were previously coded as part of the parent study.

### Statistical Methods

#### Effect Size Metrics

Continuous outcomes were measured using the small-sample corrected standardized mean difference (Hedges’ *g*; Hedges, 1981), with positive values indicating beneficial effects (e.g., greater reductions in cannabis use compared to control conditions). For the binary outcome of general cannabis use, we used log odds ratio effect sizes, with higher values indicating greater odds of *not* using cannabis compared to the control conditions. Log odds were transformed to odds ratios in narrative interpretations of those outcomes.

#### Effect Size Independence

Traditional meta-analysis models assume statistically independent error terms for the included effect sizes, and violation of this assumption, such as naively including multiple effect sizes from the same study, will produce incorrect standard errors (Higgins et al., 2024). To ensure each meta-analysis pooled a set of statistically independent effect sizes, only one effect size from each study was included in any given model. If studies reported multiple measures of cannabis use consumption levels, the following hierarchy was used for effect size selection, in order of preference: (1) frequency of use measured in days; (2) frequency of use measured in times cannabis was used over a week or longer time period; and (3) quantity of cannabis consumed. If studies reported general cannabis use for multiple recall periods, the effect size closest to the modal recall period was selected.

Some studies measured the same outcome across multiple timepoints (e.g., both 6-month and 12-month follow-up). In these cases, the effect size closest to the modal timepoint was selected, and if a modal timepoint was not present, preference was given to the earlier timepoint. Additionally, some studies included multiple intervention groups. For multi-arm studies that tested different intervention dosages and/or enhancements, the contrast between the most minimal control group and the intervention with the highest level of treatment exposure or support (i.e., the most intensive intervention) was selected. When multi-arm studies evaluated interventions delivered both in-person and electronically, effect sizes for the in-person interventions were selected as that was the most common modality in the analytic sample.

#### Analytic Strategy

First, we estimated random-effects models with the restricted maximum likelihood estimator for *τ*^2^ to examine short-term and long-term outcome results. To limit potential bias and uncertainty, the models were implemented with inverse-variance weighting, which assigns greater weight to studies with more precise estimates (Higgins et al., 2024). Because only one study measured severity at a long-term follow-up, only short-term severity results were synthesized. Next, mixed effects meta-regression models were estimated with the Knapp and Hartung adjustment to examine moderators (Knapp & Hartung, 2003). For subsequent subgroup analyses, the between studies variance (*τ*^2^) was computed within subgroups, and each estimate of *τ*^2^ was pooled and applied to all the subgroups. Forest plots were used to visually display results. All analyses were conducted in R using the metafor package (version 4.4; Viechtbauer, 2010).

#### Sensitivity Testing

In accordance with best practices, sensitivity tests were conducted to evaluate the robustness of findings (Higgins et al., 2024). To address potential biases from the decisions made for handling non-independent effect sizes, we replicated the analyses using alternative decisions for selecting effect sizes. For studies that reported multiple effect sizes for a given meta-analysis model, we assessed whether choosing an alternative follow-up timepoint, less intensive intervention contrast, or electronic modality impacted the findings. These sensitivity tests were only conducted for cannabis consumption levels and general use outcomes because none of the severity outcome analyses was affected by the initial decisions. Additionally, timepoint sensitivity tests were limited to outcomes that were reported at multiple timepoints in the included studies. Specifically, the effect size selection process described above yielded short-term effect sizes that were all measured at 3 months post-intervention, and therefore, timepoint sensitivity tests were not conducted for this timepoint. Finally, for analyses with ten or more effect sizes included, funnel plots, regression tests for funnel plot asymmetry, and trim and fill analysis were used to assess for small study bias (Egger et al., 1997; Rothstein et al., 2005).

## Results

The PRISMA flow diagram is displayed in Fig. 1. The parent meta-analysis included effect sizes from 111 studies; 73 of those studies were excluded from the present meta-analysis due to an ineligible intervention (i.e., alcohol-focused interventions). Among the remaining 38 studies, 21 were excluded due to ineligible outcomes (i.e., no cannabis outcomes reported). The remaining 17 studies were eligible for inclusion in the present meta-analysis, comprising data collected from 23 unique study samples and reported in 18 reports. As shown in Table 1, most studies took place in North America (82%). Using the Cochrane Collaboration’s risk of bias tool for randomized controlled trials, most studies were rated as having an unclear risk of bias (82%), with domains of allocation concealment, blinding of outcome assessors, and selective reporting having the greatest risk for bias (see Supplemental Material 2 for study-level risk of bias ratings). The most common delivery settings were community health centers (41%) and emergency departments (29%). Most interventions targeted mixed age groups or adults only (41%) and adolescents only (29%). Thirty-five percent of the BDIs included a booster, and only six studies (35%) specifically targeted cannabis use during the BDI. All studies included a CONSORT diagram. Study-level characteristics are reported in Table 2.

**Table 1:** Aggregate characteristics of included studies (*k* = 17)

Characteristic	*k* (%)
Geographic region	
North America	14 (82.4%)
Africa	1 (5.9%)
Europe	1 (5.9%)
Multiple regions	1 (5.9%)
Risk of bias	
*Random sequence generation*	
Low	11 (64.7%)
Unclear	6 (35.3%)
*Allocation concealment*	
Low	8 (47.1%)
Unclear	9 (52.9%)
*Blinding of outcome assessors*	
Low	1 (5.9%)
High	1 (5.9%)
Unclear	15 (88.2%)
*Selective reporting*	
Low	3 (17.6%)
High	1 (5.9%)
Unclear	13 (76.5%)
*Incomplete outcome data*	
Low	10 (58.8%)
High	1 (5.9%)
Unclear	6 (35.3%)
*Other bias*	
Low	13 (76.5%)
Unclear	4 (23.5%)
*Overall risk of bias*	
High	3 (17.6%)
Unclear	14 (82.4%)
Consort diagram	
No	0 (0.0%)
Yes	17 (100.0%)
Setting	
Community health center	7 (41.2%)
Emergency department	5 (29.4%)
Multiple	2 (11.8%)
University health center	2 (12.8%)
Hospital-based primary care	1 (5.9%)
Population	
Mixed ages and adults only	7 (41.2%)
Adolescents only	5 (29.4%)
Adolescents and young adults	3 (17.6%)
University students	2 (11.8%)
Booster session	
No	11 (64.7%)
Yes	6 (35.3%)
Intervention target	
Other drug/mixed substances	11 (64.7%)
Cannabis	6 (35.3%)

**Table 2:** Characteristics of included studies (*k* = 17)

Study	Outcome domain(s)	Modality	Risk of Bias	Population	Setting	Target	Booster	FU timepoint (months)	Intensity
Bernstein et al. (2009)	Consumption level	In-person	Unclear	Adolescents and YA	ED	Cannabis	Y	3, 12	N
Blow et al. (2017)	Consumption level	*Electronic*, in-person	High	Mixed ages and adults only	ED	OD/MS	Y	3, 6, *12*	*Y*
D’Amico et al. (2008)	Consumption level	In-person	Unclear	Adolescents	CHC	OD/MS	Y	3	N
D’Amico et al. (2018)	Consumption level	In-person	Unclear	Adolescents	CHC	OD/MS	N	3, 6, *12*	N
Goodness and Palfai (2020)	Consumption level	Electronic	Unclear	University students	UHC	Cannabis	Y	3, 6	N
Gryczynski et al. (2016)	Use, severity	Electronic	Unclear	Mixed ages and adults only	CHC	OD/MS	N	3	N
Humeniuk et al. (2012)*	Severity	In-person	High	Mixed ages and adults only	Multiple	OD/MS	N	3	N
Knight et al. (2019)**	Use	In-person	Unclear	Adolescents	Multiple	OD/MS	N	12	N
Laporte et al. (2017)	Consumption level	In-person	Unclear	Adolescents and YA	CHC	Cannabis	N	3, 6, *12*	N
Mason et al. (2015)	Consumption level	In-person	Unclear	Adolescents	CHC	OD/MS	N	3, 6	N
Merchant et al. (2015)	Use, severity	In-person	Unclear	Mixed ages and adults only	ED	OD/MS	Y	3	N
Mertens et al. (2014)	Severity	In-person	Unclear	Adolescents and YA	CHC	OD/MS	N	3	N
Palfai et al. (2014)	Consumption level	Electronic	Unclear	University students	UHC	Cannabis	N	3, 6	N
Saitz et al. (2014)	Use, severity	In-person	Unclear	Mixed ages and adults only	HPC	OD/MS	Y	6	*Y*
Walsh et al. (2017)	Consumption level	Electronic	High	Mixed ages and adults only	ED	OD/MS	N	3, 6	N
Walton et al., (2013, 2014)**	Consumption level, use	*Electronic*, in-person	Unclear	Adolescents	CHC	Cannabis	N	3, 6, *12*	N
Woolard et al. (2013)	Consumption level	In-person	Unclear	Mixed ages and adults only	ED	Cannabis	N	3, 6	N

Effect sizes associated with italicized characteristics were only included in the respective sensitivity analyses

FU*,* follow-up; CHC, community health center; ED*,* emergency department; HPC*,* hospital-based primary care; UHC*,* university health center; YA, young adults; OD/MS, other drugs/mixed substances

*Reported outcomes for four independent samples based on recruitment sites

**Reported outcomes for two independent samples based on cannabis initiation status

### Main Effects

Standardized effect sizes and heterogeneity statistics are reported in Table 3 for all estimable time periods and outcomes. Long-term severity effects could not be synthesized because only one eligible study measured cannabis use severity during that follow-up period. Forest plots from these models are also displayed in Supplemental Material 3.

**Table 3:** Standardized effect sizes, 95% confidence intervals, and heterogeneity statistics by outcome domain and time period

	**0–5 months**				**6–12 months**			
Cannabis outcome	ES [95% CI]	[95% PI]	*τ*^2^	*I*^2^	ES [95% CI]	[95% PI]	*τ*^2^	*I*^2^
Consumption level	0.01 [− 0.07, 0.09]_12_	[− 0.07, 0.09]	0.00	0.00%	0.04 [− 0.05, 0.12]_11_	[− 0.07, 0.15]	0.00	5.15%
Use	0.19 [− 0.11, 0.48]_3_	[− 0.11, 0.48]	0.00	0.00%	0.18 [− 0.32, 0.67]_4_	[− 0.76, 1.11]	0.16	63.85%
Severity	0.13 [− 0.07, 0.33]_7_	[− 0.31, 0.57]	0.03	63.73%	-	-	-	-

Subscripts denote the number of independent effect sizes included for each outcome; consumption level and severity effect sizes are Hedges’ *g* and use effect sizes are logORs

ES, effect size; CI, 95% confidence intervals; PI, 95% prediction intervals

#### Cannabis Consumption Level

Relative to practice as usual controls, there was no evidence of BDI effects on short-term (*k* = 12, *g* = 0.01, 95% CI [− 0.07, 0.09], *p* = 0.802) or long-term (*k* = 11, *g* = 0.04, 95% CI [**− **0.05, 0.12], *p* = 0.413) cannabis consumption levels. In other words, there was less than a 0.05 standard deviation difference in reported cannabis consumption levels between the BDI and control groups during both time periods. Indeed, although 13 of the 23 total effect sizes for this outcome were positive, none was statistically significant. There was no evidence of between-study heterogeneity for the short-term (*τ*^2^ = 0.00, *I*^2^ = 0.00%) or long-term (*τ*^2^ = 0.03, *I*^2^ = 5.15%) outcomes.

#### Cannabis Use

There was also no evidence of BDI effects on short-term (*k* = 3, logOR = 0.19, 95% CI [− 0.11, 0.48], *p* = 0.217) or long-term (*k* = 4, logOR = 0.18, 95% CI [− 0.32, 0.67], *p* = 0.488) outcomes of any cannabis use. Interpreted another way, the odds of *not* using cannabis were approximately 20% higher for participants assigned to BDIs versus those assigned to the control groups, but these differences were not statistically significant. Although there was no evidence of heterogeneity in any cannabis use at the short-term follow-up (*τ*^2^ = 0.00, *I*^2^ = 0.00%), there was evidence of heterogeneity at long-term follow-up (*Q*_(3)_ = 8.61, *τ*^2^ = 0.16%, *p* = 0.035), with 63.9% of the observed heterogeneity reflecting variance in true effects rather than sampling error. Thus, considerable variation in the long-term odds of not using cannabis could be expected in future BDI trials (95% PI = [0.47, 3.04]).

#### Cannabis Use Severity

Synthesis of seven independent effect sizes (81% positive) from four studies also showed no evidence of BDI effects on short-term cannabis use severity (*g* = 0.13, 95% CI [− 0.07, 0.33], *p* = 0.199). There was minimal heterogeneity (*τ*^2^ = 0.04, *Q*_(6)_ = 13.39, *p* = 0.037), with 63.7% of the observed heterogeneity reflecting variance in true effects rather than sampling error. Thus, considerable variation in short-term effects on cannabis severity could be expected in future BDI trials (95% PI = [− 0.31, 0.57]).

### Moderation Analysis

Subgroup effect sizes and 95% confidence intervals for the moderation analyses are reported in Table 4. Although short-term use outcomes are reported for completeness, the results should be interpreted with caution due to large standard errors and limited degrees of freedom. Overall, there was no evidence that effects of BDIs were moderated by booster session status (*F* = 1.12, *p* = 0.367), setting (*F* = 3.02, *p* = 0.094), intervention target (*F* = 3.05, *p* = 0.745), or population (*F* = 1.40, *p* = 0.326). Moreover, none of the subgroups was associated with significant changes in cannabis outcomes during either follow-up period.

**Table 4:** Subgroup effect sizes and 95% confidence intervals by outcome domain and follow-up period

	**0–5 months**			**6–12 months**	
**Subgroup**	**Consumption level**	**Use**	**Severity**	**Consumption level**	**Use**
Booster session					
No	− 0.02 [− 0.11, 0.08]_8_	− 0.11 [− 5.93, 5.71]_1_	0.15 [− 0.17, 0.47]_6_	− 0.01 [− 0.10, 0.12]_8_	0.39 [− 0.59, 1.37]_1_
Yes	0.14 [− 0.07, 0.35]_4_	0.24 [− 5.27, 5.75]_2_	0.09 [− 0.55, 0.72]_1_	0.16 [− 0.08, 0.41]_3_	− 0.33 [− 1.61, 0.94]_3_
Setting					
Community health center	− 0.02 [− 0.15, 0.10]_6_	− 0.11 [− 5.93, 5.71]_2_	− 0.11 [− 0.52, 0.74]_2_	− 0.06 [− 0.17, 0.06]_5_	0.00 [− 3.89, 3.89]_1_
Emergency department	0.01 [− 0.14, 0.17]_4_	0.24 [− 5.27, 5.75]_1_	0.09 [− 0.70, 0.88]_1_	0.13 [− 0.00, 0.27]_4_	-
Hospital-based primary care	-	-	-	-	− 0.33 [− 2.86, 2.20]_1_
Multiple settings	-	-	0.18 [− 0.20, 0.66]_4_	-	0.57 [− 2.09, 3.23]_2_
University health center	0.22 [− 0.11, 0.55]_2_	-	-	0.21 [− 0.09, 0.51]_2_	-
Target					
Other drugs/mixed substances	0.07 [− 0.09, 0.23]_6_	− 0.04 [− 5.83, 5.75]_2_	-	0.05 [− 0.15, 0.24]_5_	0.25 [− 1.21, 1.70]_3_
Cannabis	− 0.02 [− 0.13, 0.10]_6_	0.20 [− 7.85, 8.26]_1_	-	0.04 [− 0.11, 0.18]_6_	− 0.00 [− 2.62, 2.62]_1_
Population					
Adolescents only	0.01 [− 0.12, 0.14]_5_	0.20 [−7.85, 8.26]_1_	0.07 [− 0.58, 0.72]_1_	− 0.05 [− 0.20, 0.10]_4_	0.39 [− 0.59, 1.37]_3_
Adolescents and young adults	− 0.15 [− 0.42, 0.11]_2_	-	-	0.01 [− 0.28, 0.30]_2_	-
Mixed and adults only	0.02 [− 0.13, 0.18]_3_	− 0.04 [− 5.83, 5.75]_2_	0.15 [− 0.16, 0.46]_6_	0.13 [− 0.05, 0.31]_3_	− 0.33 [− 1.61, 0.94]_1_
University students	0.22 [− 0.11, 0.55]_2_	-	-	0.21 [− 0.14, 0.56]_2_	-

Subscripts denote the number of independent effect sizes in each subgroup; consumption level and severity effect sizes are Hedges’ *g* and use effect sizes are logORs

### Sensitivity Tests

**Fig. 2:**
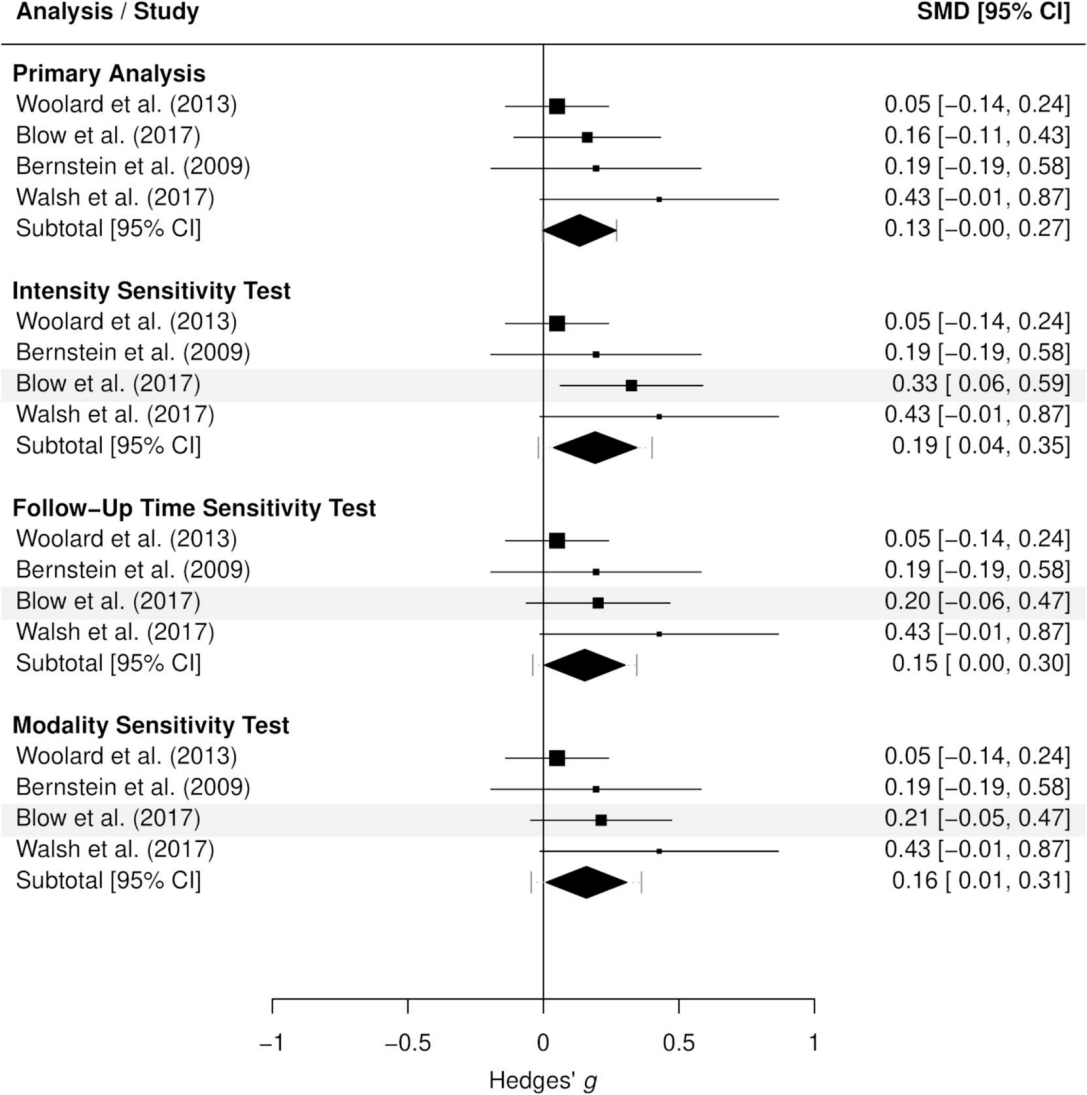
Long-term cannabis consumption level results for emergency department subgroups. Forest plot illustrating individual study effects (squares, with size representing weight), pooled subgroup effect estimates (diamonds), and prediction intervals (dashed lines extending from diamond). Highlighted study effects differed from those included in the primary analytic sample. SMD, Hedges’ *g* standardized mean difference

The main effect findings from the sensitivity tests were consistent with those of the primary analyses reported above (full results are reported in Supplemental Material 4). The sensitivity tests for the moderator analyses findings were also generally consistent with the primary findings reported above, with one notable exception. BDIs implemented in emergency departments evidenced small but significant reductions in cannabis use consumption levels when selecting alternative effect sizes based on follow-up timepoint (*g* = 0.15 [0.00+, 0.30], *p* = 0.049), intervention intensity (*g* = 0.19 [0.04, 0.35], *p* = 0.021), and modality (*g* = 0.16 [0.01, 0.31], *p* = 0.042). As shown by the forest plot in Fig. 2, these selection choices resulted in a higher effect size for this subgroup than obtained from the primary analytic sample (*g* = 0.13 [− 0.00, 0.27], *p* = 0.054). Full moderation results for the sensitivity tests are reported in Supplemental Material 5.

## Discussion

This meta-analysis had two aims: to (1) evaluate the effects of BDIs delivered in general medical settings on cannabis outcomes; and (2) assess whether those effects varied by booster session status, intervention target, setting, and population. Overall, we found no evidence of consistently beneficial or harmful effects of BDIs on cannabis use consumption levels, general cannabis use, or severity of use at both short-term and long-term follow-up periods. There was also no evidence that these effects were moderated by booster session status, intervention target, setting, or population. These findings were generally consistent across a variety of sensitivity tests but may be admittedly conservative. Nonetheless, the results do not suggest that implementing BDIs in general medical settings would lead to consistent reductions in medical patients’ cannabis consumption or severity.

Findings from the sensitivity analyses, however, suggest that there is a subgroup for whom BDIs may lead to beneficial long-term effects on cannabis outcomes. In each of the three sensitivity tests, BDIs delivered in emergency departments were associated with significant reductions in cannabis consumption levels, providing secondary evidence that BDIs may perform more favorably when delivered in emergency departments compared to general medical settings. This is consistent with a recent review that found that only BDIs delivered in emergency departments had significant reductions in drug use frequency (Sahker et al., 2022) and suggests that BDIs may be better suited for implementation in that specific setting. These more favorable results may stem from the high prevalence of emergency department visits attributable to substance use, with cannabis being the second most common illicit drug involved in emergency department admissions (SAMHSA, 2023a). Individuals receiving care in an emergency department may have more severe clinical presentations and thus benefit more from a single BDI. Additionally, if the emergency department visit stemmed from cannabis or other drug-related consequences (e.g., cannabinoid hyperemesis syndrome and vehicle accidents), participants may have greater motivation to make behavioral changes and thus be better candidates for BDIs (Hawk & D’Onofrio, 2018).

Nevertheless, the lack of significant main effects aligns with findings from other meta-analyses evaluating BDIs delivered in healthcare settings (Imtiaz et al., 2020; Tanner-Smith et al., 2022). Similarly, other trials have reported no evidence of reductions in cannabis outcomes after delivering brief interventions in healthcare settings (Bernstein et al., 2010; Cancilliere et al., 2018; Poblete et al., 2017). Brief interventions remain a compelling approach to reducing substance use and related consequences given their brevity and low cost, consistent evidence of beneficial effects on reducing alcohol use, and importance as a first step along the continuum of care (Halladay et al., 2019). BDIs, in particular, may be integral in linking individuals to subsequent substance use treatment (i.e., referrals to treatment); prior studies have found that participants who received BDIs were significantly more likely to attend substance use treatment during the follow-up periods than participants in the comparison groups (Krupski et al., 2010; Tait et al., 2004). Other specialized treatment centers or alternative community settings may show differential effectiveness of BDIs, where patients present with more or less severe illicit drug use behaviors and where they may be more motivated or prepared to make behavioral changes. However, our findings clearly warrant consideration of *how* BDIs delivered in general medical settings, specifically, can be improved upon to address cannabis consumption and severity. Another possibility is that BDIs targeting cannabis use are differentially effective for patients with different identities or presenting characteristics. For example, Schweer-Collins and colleagues (2023) explored whether BDIs varied according to patient sex, age, housing status, relationship status, education level, and baseline severity of drug or alcohol use using individual patient data; however, no evidence of variability was found for any cannabis- or drug-use-related outcomes. Similar to the current study, there were few studies exploring the outcomes of drug-specific BDIs (*k* = 2–5); thus, future trials should continue to explore the populations for whom and settings under which BDIs targeting cannabis use may be more or less effective.

### Limitations

Although this meta-analysis fills important gaps in the knowledge base around BDIs, its findings should be viewed in light of its limitations. First, this meta-analysis synthesized findings from a small number of trials in several subgroup analyses. As a result, these subgroup results should be interpreted and generalized with caution. Relatedly, given the small number of studies evaluating the effects of BDIs on cannabis use outcomes, our ability to assess and explain heterogeneity was inherently limited. Second, none of the included studies reported cannabis outcomes collected after 12 months post-intervention. Therefore, our findings may not generalize to longer follow-up periods. Third, both biomarkers and self-report instruments were used to measure cannabis use in the included studies. The validity of self-report instruments in BDI trials is a concern due to social desirability bias and potential underreporting of illicit substance use (Saitz, 2014), which may contribute to differences in intervention effects across studies. Finally, all synthesized studies included in this meta-analysis were rated as having unclear or high overall risk of bias. Thus, it is unknown how these findings would change if they were conducted with a lower risk of bias.

### Future Considerations

As cannabis use continues to evolve in the wake of medical and recreational legalization, it may be beneficial for BDIs targeting people who use cannabis to include more harm reduction strategies. This might include content about using lower-potency cannabis products and adopting alternative routes of use over smoking cannabis to reduce pulmonary health risks (Fischer et al., 2022). Delaying the onset of cannabis use to adulthood has been identified as a harm reduction strategy that may reduce the risk of adverse health outcomes (Fischer et al., 2022), but only two of the trials included in this meta-analysis tested BDIs among youth who had never used cannabis (Knight et al., 2019; Walton et al., 2014). Thus, youth-focused preventive BDIs delivered in general medical settings warrant further exploration, with particular attention to their developmental appropriateness to ensure maximal impact (Nation et al., 2003).

In addition, further research is needed to expand and improve upon the current body of evidence. Given the findings from our sensitivity analyses, additional high-quality trials evaluating the effects of BDIs in emergency department settings are needed. Trials should also be conducted in urgent care centers and free-standing emergency departments to evaluate whether effects may vary across different models of emergency medical treatment. Moreover, BDIs implemented in other settings may have greater beneficial effects on cannabis outcomes. For example, a meta-analysis that synthesized the effects of cannabis-targeted brief interventions delivered in school, healthcare, truancy centers, and general community settings found that youth receiving interventions had higher rates of cannabis abstinence and lower cannabis use disorder symptoms versus those assigned to control groups (Halladay et al., 2019). As such, researchers should continue to synthesize the evidence of BDI trials in other settings to further assess this. Given recent reviews highlighting the importance of the duration and components (e.g., motivational interviewing and personalized feedback) of cannabis-targeted brief interventions (Gex et al., 2024; Parmar & Sarkar, 2017), future meta-analyses should include these characteristics as potential candidate moderators. Finally, more effort should be placed into developing and testing alternative prevention and intervention approaches to comprehensively address cannabis use as well as increasing access to and strengthening referral systems for substance use treatment.

## Conclusion

The current study’s findings do not lend meaningful support for the *overall* effectiveness of BDIs on cannabis consumption or severity when delivered in general medical settings. However, they provide preliminary, suggestive evidence that BDIs may perform more favorably when delivered in emergency departments. Given the current body of evidence and growing trend in cannabis use, more high-quality trials evaluating the effects of BDIs—particularly those implemented in emergency medical settings—are clearly needed.

## Supplementary Information

Below is the link to the electronic supplementary material.

Supplementary file1 (DOCX 31 KB)

Supplementary file2 (DOCX 69 KB)

Supplementary file3 (DOCX 1027 KB)

Supplementary file4 (DOCX 27 KB)

Supplementary file5 (DOCX 31 KB)

## Data Availability

Details of the data and how to request access are available from the parent study’s Principal Investigator, Emily Tanner-Smith.

## Declarations

### Protocol Registration

Protocol for the parent systematic review and meta-analysis was pre-registered in PROSPERO (CRD42018086832). The analytic plan for this review was pre-registered in OSF: https://osf.io/c82vg.
