# Supplementary material for: Brief Drug Interventions Delivered in General Medical Settings: a Systematic Review and Meta-analysis of Cannabis Use Outcomes
Source: Prev Sci. 2025 Jul 8;26(6):985–98. doi: 10.1007/s11121-025-01826-7 (PMC12394317; doi:10.1007/s11121-025-01826-7)
Supplement: Supplementary file 1 — Supplementary file1 (DOCX 31 KB) [file 11121_2025_1826_MOESM1_ESM.docx]

**Supplemental Material S1: Synthesized Outcome Measures**

| **Table 1**  *Descriptions of Outcome Measures* | | | |
| --- | --- | --- | --- |
| ***Cannabis Consumption Level*** | | | |
| **Study** | **Outcome** | **Instrument** | **Description** |
| Bernstein et al. (2009) | Days of cannabis use (past 30 days) | Timeline Follow-Back (TLFB; Sobell & Sobell, 1992) | The TLFB is a retrospective calendar-based measure of substance use over a specified time period. It has demonstrated strong reliability and validity in measuring cannabis use (Robinson et al., 2014), including high agreement with biological measures (Hjorthøj et al., 2012). |
| Blow et al. (2017) | Days of cannabis use (past 90 days) | TLFB (Sobell & Sobell, 1992) | The TLFB is a retrospective calendar-based measure of substance use over a specified time period. It has demonstrated strong reliability and validity in measuring cannabis use (Robinson et al., 2014), including high agreement with biological measures (Hjorthøj et al., 2012). |
| D'Amico et al. (2008) | Days of cannabis use (past month) | RAND Adolescent/Young Adult Panel Survey (Tucker et al., 2003) | Single item assessing the number of days cannabis was used in the past month. Participants reported days of cannabis use on a 6-point Likert scale (0 = none, 5 = 10 or more days). |
| D'Amico et al. (2018) | Number of times cannabis was used (past three months) | Adapted from the National Longitudinal Study of Adolescent Health (Sieving et al., 2001) and Ellickson et al. (2003) | Single item assessing the number of times cannabis was used in the past three months. Participants reported occurrence on a 6-point Likert scale (0 = none, 5 = 20 or more times). |
| Goodness & Palfai (2020) | Days of cannabis use (past 90 days) | Adapted from Lee et al. (2013) | Single item assessing the number of days cannabis was used in the past 90 days. The item was adapted from Lee et al. (2013) as a modified TLFB measure. Three-month calendars were provided to support recall accuracy. |
| Laporte et al. (2017) | Number of joints consumed per month | Not reported | N/A |
| Mason et al. (2015) | Days of cannabis use (past month) | Youth Risk Behavior Surveillance Survey (Centers for Disease Control and Prevention, 2008) | Single item assessing the number of days cannabis was used in the past month. Participants reported days of cannabis use on an 8-point Likert scale (0 = 0 days, 7 = all 30 days). |
| Palfai et al. (2014) | Days of cannabis use (past 90 days) | Adapted from Lee et al. (2013) | Single item assessing the number of days cannabis was used in the past 90 days. The item was adapted from Lee et al. (2013) as a modified TLFB measure. Three-month calendars were provided to support recall accuracy. |
| Walsh et al. (2017) | Days of cannabis use (past 14 days) | Not reported | N/A |
| Walton et al. (2013); Walton et al. (2014) | Days of cannabis use (past three months) | Adapted from the National Longitudinal Study of Adolescent Health (Sieving et al., 2001) | Single item assessing the number of days cannabis was used in the past three months. Participants reported days of cannabis use on a 7-point Likert scale (0 = never, 6 = every day or almost every day). |
| Woolard et al. (2013) | Days tetrahydrocannabinol (THC) use (past 30 days) | Alcohol, Marijuana, and Drug Use Index (Nirenberg, & Lee, 2003) | Single item measuring days of cannabis (i.e., THC) use was the outcome of interest. |
| ***Cannabis Use*** | | | |
| **Study** | **Outcome** | **Instrument** | **Description** |
| Saitz et al. (2014) | Positive hair test indicating cannabis use | Detection of carboxy-THC in hair sample | Participants were provided a hair sample corresponding to a 90-day timeframe and tested using enzyme-linked immunosorbent assay and gas chromatography–mass spectrometry. |
| Merchant et al. (2015) | Any cannabis use or misuse (past three months) | The Alcohol, Smoking and Substance Involvement Screening Test (ASSIST; WHO ASSIST Working Group, 2002) | Dichotomized measure derived from ASSIST item assessing how often cannabis was non-medically used in the past three months. The cannabis-specific items have demonstrated strong reliability and validity (Khazaal et al., 2015; WHO ASSIST Working Group, 2002). |
| Gryczynski et al. (2016) | Positive hair test indicating cannabis use | Detection of carboxy-THC in hair sample | Participants provided a 1.5” hair sample (corresponding to a 3-month timeframe). The sample was tested for carboxy-THC by an independent laboratory. |
| Knight et al. (2019) | Any cannabis use (past 12 months) | TLFB (Sobell & Sobell, 1992) | Dichotomized measure derived from TLFB measure of cannabis use over past 12 months. The TLFB has demonstrated strong reliability and validity in measuring cannabis use (Robinson et al., 2014), including high agreement with biological measures (Hjorthøj et al., 2012). |
| Walton et al. (2013); Walton et al. (2014) | Any cannabis use (past three months) | Adapted from the National Longitudinal Study of Adolescent Health (Sieving et al., 2001) | Dichotomized measure derived from item assessing cannabis use frequency. |
| ***Cannabis Use Severity*** | | | |
| **Study** | **Outcome** | **Instrument** | **Description** |
| Gryczynski et al. (2016)  Humeniuk et al. (2012)  Merchant et al. (2015)  Mertens et al. (2014) | Cannabis score from the Alcohol, Smoking and Substance Involvement Screening Test (ASSIST) | ASSIST (WHO ASSIST Working Group, 2002) | The ASSIST is a commonly used instrument for measuring substance use severity. It includes seven cannabis-specific items: (1) lifetime non-medical cannabis use; (2-7) if any use was reported, participants are asked past 3-month frequency of use, urgency of use, and related consequences as well as any past attempts to control use and concerns about use. The latter six items are summed to create a total score, with cutoffs indicating low (0-3), moderate (4-26), and high (27+) levels of use. The cannabis-specific items have demonstrated strong reliability and validity (Khazaal et al., 2015; WHO ASSIST Working Group, 2002). The full instrument and scoring instruction are reported in Humeniuk et al. (2010). |

**References**

Centers for Disease Control and Prevention. (2008). 2007 Youth Risk Behavior Surveillance Survey. www.cdc.gov/yrbss

Ellickson, P. L., McCaffrey, D. F., Ghosh-Dastidar, B., & Longshore, D. L. (2003). New inroads in preventing adolescent drug use: Results from a large-scale trial of Project ALERT in middle schools. *American Journal of Public Health*, *93*(11), 1830–1836. https://doi.org/10.2105/AJPH.93.11.1830

Hjorthøj, C. R., Hjorthøj, A. R., & Nordentoft, M. (2012). Validity of Timeline Follow-back for self-reported use of cannabis and other illicit substances—Systematic review and meta-analysis. *Addictive Behaviors*, *37*(3), 225–233. https://doi.org/10.1016/j.addbeh.2011.11.025

Humeniuk, R., Henry-Edwards, S., Ali, R., Poznyak, V., Monteiro, M. G., & World Health Organization. (2010). *The Alcohol, Smoking and Substance Involvement Screening Test (ASSIST): Manual for use in primary care*. World Health Organization. https://iris.who.int/handle/10665/44320

Khazaal, Y., Chatton, A., Monney, G., Nallet, A., Khan, R., Zullino, D., & Etter, J.-F. (2015). Internal consistency and measurement equivalence of the cannabis screening questions on the paper-and-pencil face-to-face ASSIST versus the online instrument. *Substance Abuse Treatment, Prevention, and Policy*, *10*(1), 8. https://doi.org/10.1186/s13011-015-0002-9

Lee, C. M., Kilmer, J. R., Neighbors, C., Atkins, D. C., Zheng, C., Walker, D. D., & Larimer, M. E. (2013). Indicated prevention for college student marijuana use: A randomized controlled trial. *Journal of Consulting and Clinical Psychology*, *81*(4), 702–709. https://doi.org/10.1037/a0033285

Nirenberg T, Lee C. (2003). Alcohol, Marijuana and Drug Use Index: Unpublished survey.

Robinson, S. M., Sobell, L. C., Sobell, M. B., & Leo, G. I. (2014). Reliability of the Timeline Followback for cocaine, cannabis, and cigarette use. *Psychology of Addictive Behaviors*, *28*(1), 154–162. https://doi.org/10.1037/a0030992

Sieving, R. E., Beuhring, T., Resnick, M. D., Bearinger, L. H., Shew, M., Ireland, M., & Blum, R. W. (2001). Development of adolescent self-report measures from the National Longitudinal Study of Adolescent Health. *Journal of Adolescent Health*, *28*(1), 73–81. https://doi.org/10.1016/S1054-139X(00)00155-5

Sobell, L. C., & Sobell, M. B. (1992). Timeline Follow-back. In R. Z. Litten & J. P. Allen (Eds.), *Measuring alcohol consumption: Psychosocial and biochemical methods* (pp. 41–72). Humana Press. https://doi.org/10.1007/978-1-4612-0357-5_3

Tucker, J. S., Orlando, M., & Ellickson, P. L. (2003). Patterns and correlates of binge drinking trajectories from early adolescence to young adulthood. *Health Psychology*, *22*(1), 79–87. https://doi.org/10.1037/0278-6133.22.1.79

WHO ASSIST Working Group. (2002). The Alcohol, Smoking and Substance Involvement Screening Test (ASSIST): Development, reliability and feasibility. *Addiction*, *97*(9), 1183–1194. https://doi.org/10.1046/j.1360-0443.2002.00185.x
