# Supplementary material for: Brief Drug Interventions Delivered in General Medical Settings: a Systematic Review and Meta-analysis of Cannabis Use Outcomes
Source: Prev Sci. 2025 Jul 8;26(6):985–98. doi: 10.1007/s11121-025-01826-7 (PMC12394317; doi:10.1007/s11121-025-01826-7)
Supplement: Supplementary file 2 — Supplementary file2 (DOCX 69 KB) [file 11121_2025_1826_MOESM2_ESM.docx]

**Supplemental Material S2: Risk of Bias Assessment**

| **Table 1**  *Risk of Bias Ratings for the Included Studies (k = 17)* | | | | | | | |
| --- | --- | --- | --- | --- | --- | --- | --- |
| **Study** | **Random Sequence Generation** | **Allocation Concealment** | **Blinding of Outcome Assessment** | **Incomplete Outcome Data** | **Selective Reporting** | **Other Bias** | **Overall Bias** |
| Bernstein et al. (2009) | Low | Low | Unclear | Unclear | Unclear | Low | Unclear |
| Blow et al. (2017) | Low | Unclear | Low | Unclear | High | Low | High |
| D’Amico et al. (2008) | Unclear | Unclear | Unclear | Low | Unclear | Low | Unclear |
| D’Amico et al. (2018) | Low | Low | Unclear | Low | Low | Low | Unclear |
| Goodness & Palfai (2020) | Unclear | Unclear | Unclear | Low | Unclear | Unclear | Unclear |
| Gryczynski et al. (2016) | Unclear | Low | Unclear | Unclear | Unclear | Unclear | Unclear |
| Humeniuk et al. (2011) | Low | Unclear | High | Unclear | Unclear | Low | High |
| Knight et al. (2019) | Low | Low | Unclear | Low | Unclear | Low | Unclear |
| Laporte et al. (2017) | Low | Low | Unclear | Unclear | Unclear | Low | Unclear |
| Mason et al. (2015) | Low | Unclear | Unclear | Low | Unclear | Low | Unclear |
| Merchant et al. (2015) | Low | Unclear | Unclear | Low | Low | Low | Unclear |
| Mertens et al. (2014) | Unclear | Low | Unclear | Unclear | Unclear | Low | Unclear |
| Palfai et al. (2014) | Unclear | Unclear | Unclear | Low | Unclear | Unclear | Unclear |
| Saitz et al. (2014) | Low | Low | Unclear | Low | Low | Low | Unclear |
| Walsh et al. (2017) | Low | Unclear | Unclear | High | Unclear | Unclear | High |
| Walton et al. (2013, 2014) | Low | Unclear | Unclear | Low | Unclear | Low | Unclear |
| Woolard et al. (2013) | Unclear | Low | Unclear | Low | Unclear | Low | Unclear |

| **Figure 1**  *Risk of Bias Traffic Light Plot* | | | | | | | |
| --- | --- | --- | --- | --- | --- | --- | --- |
|  | **Risk of Bias** | | | | | | |
| **Study** | **D1** | **D2** | **D3** | **D4** | **D5** | **D6** | **Overall** |
| Bernstein et al. (2009) | 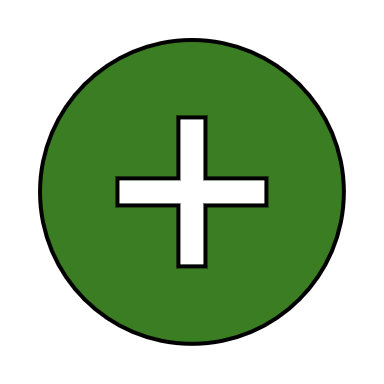 | 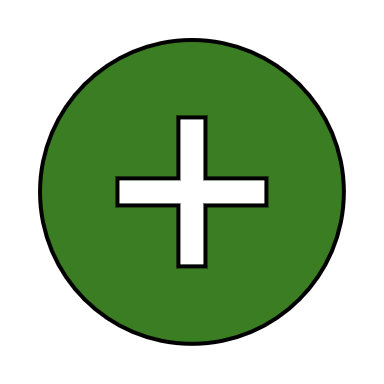 | 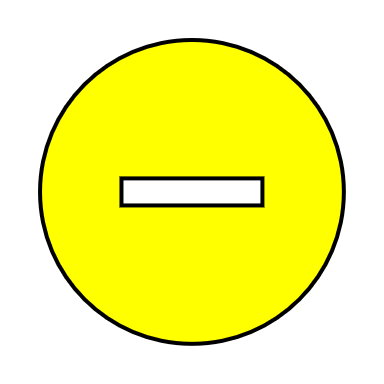 | 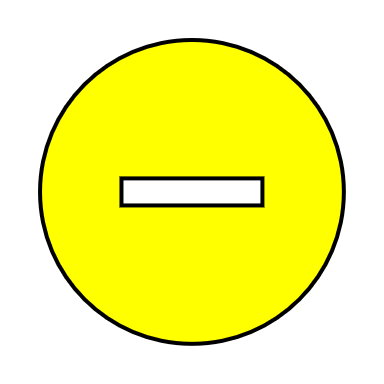 | 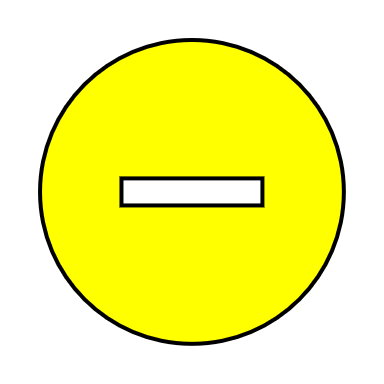 | 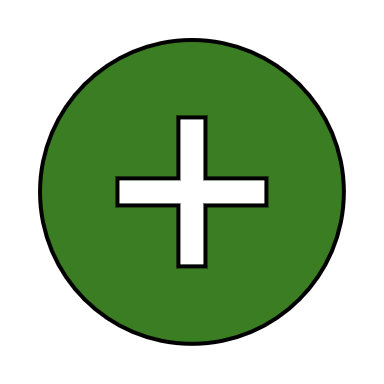 | 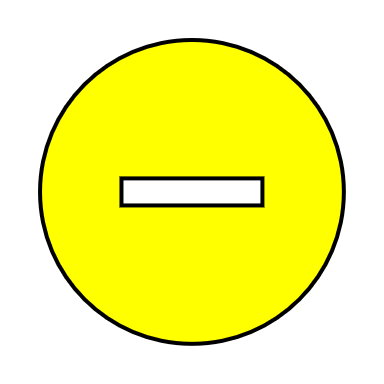 |
| Blow et al. (2017) | 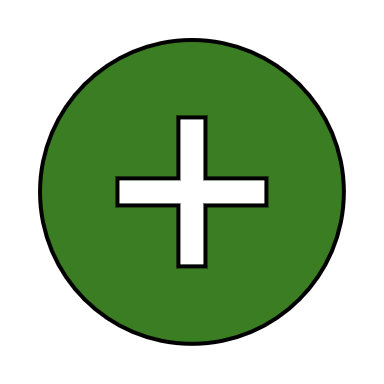 | 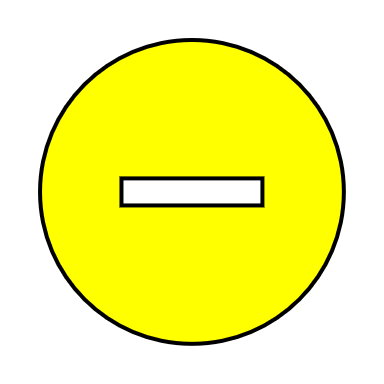 | 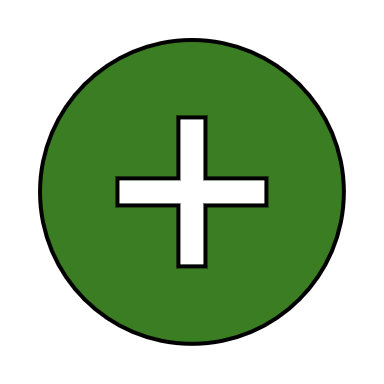 | 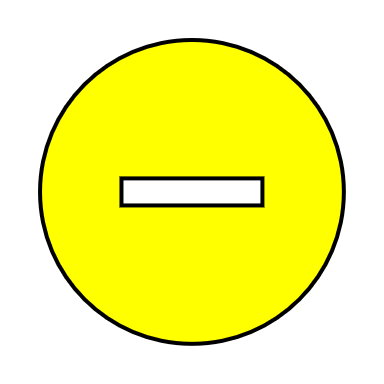 | 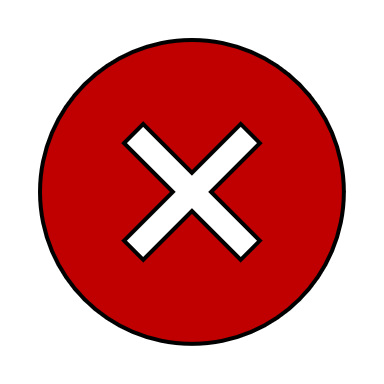 | 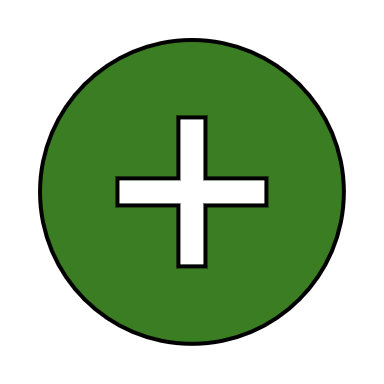 | 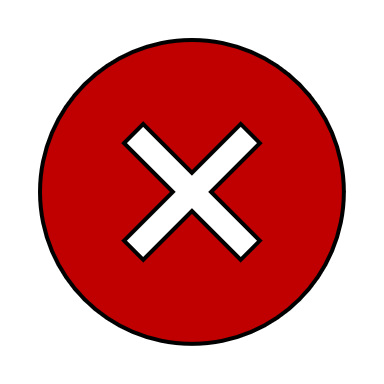 |
| D'Amico et al. (2008) | 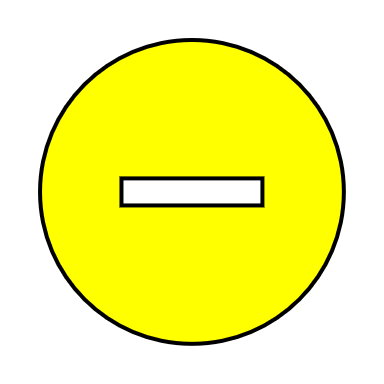 | 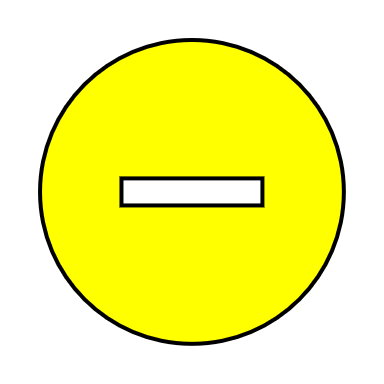 | 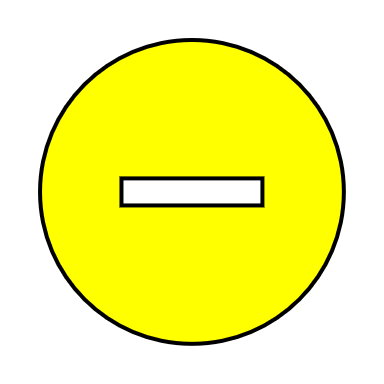 | 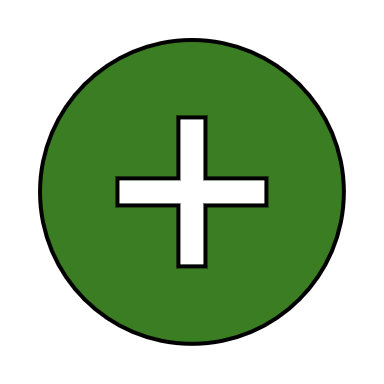 | 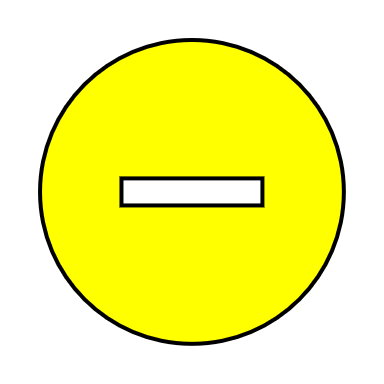 | 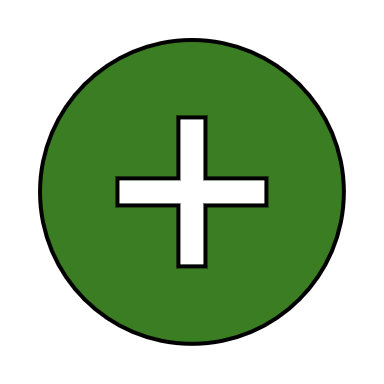 | 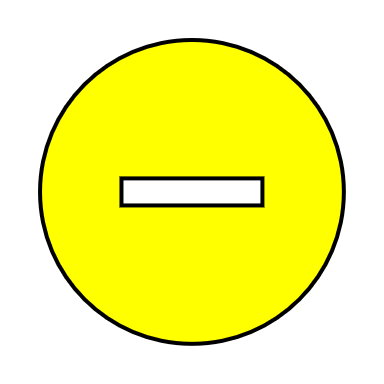 |
| D'Amico et al. (2018) | 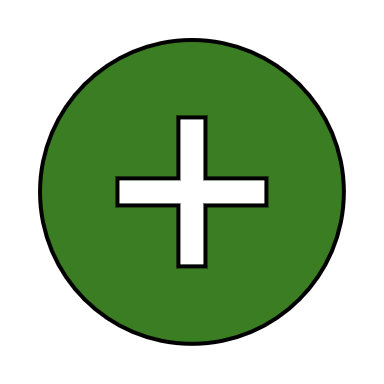 | 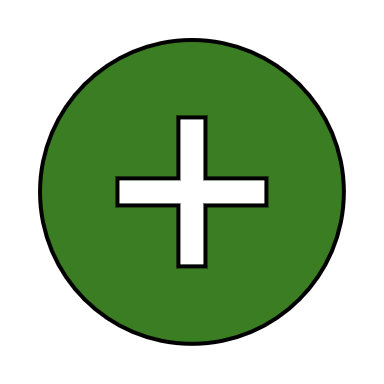 | 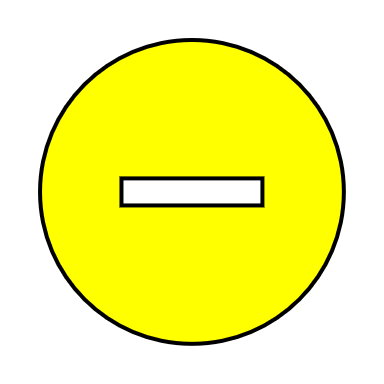 | 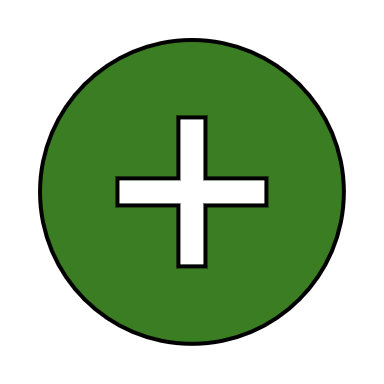 | 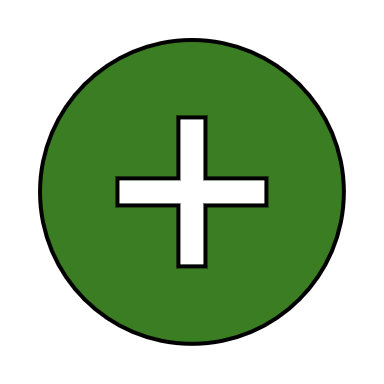 | 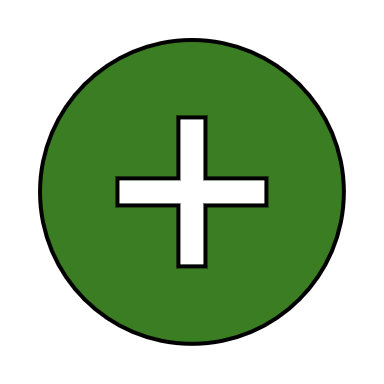 | 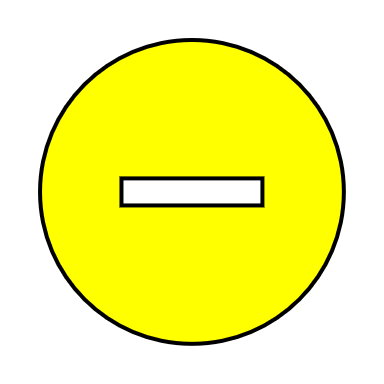 |
| Goodness & Palfai (2020) | 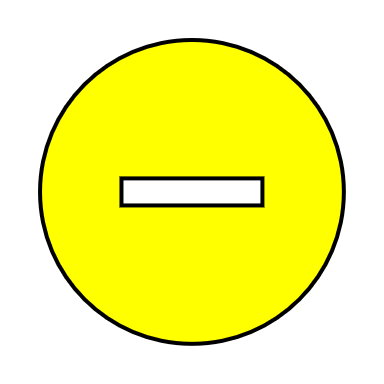 | 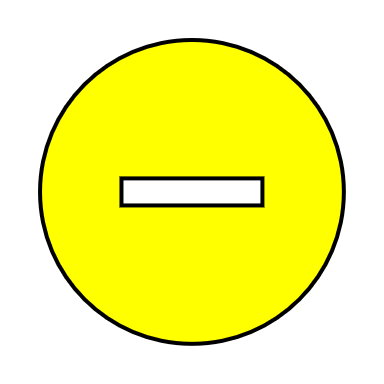 | 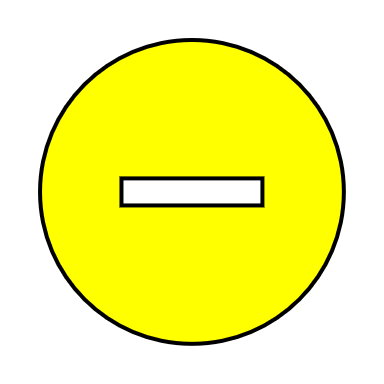 | 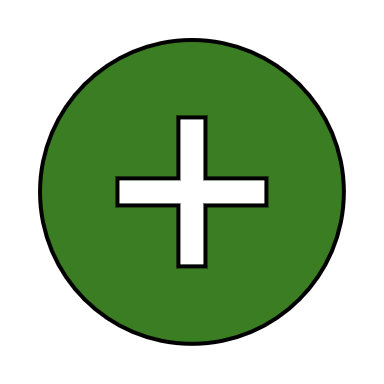 | 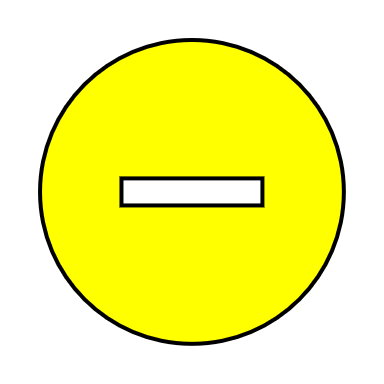 | 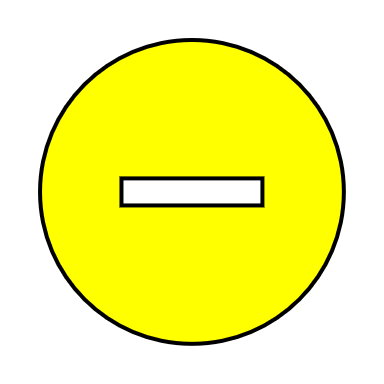 | 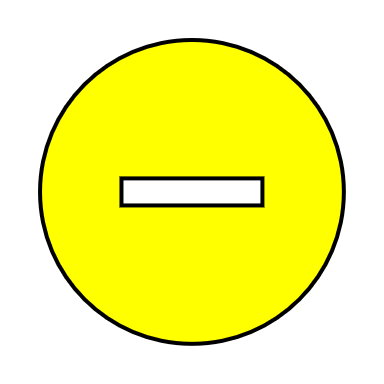 |
| Gryczynski et al. (2016) | 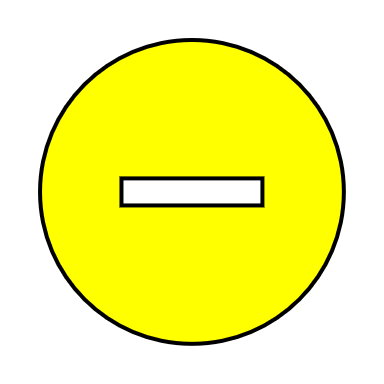 | 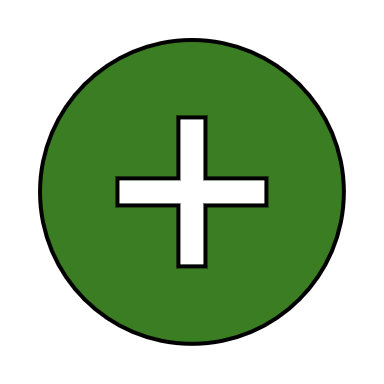 | 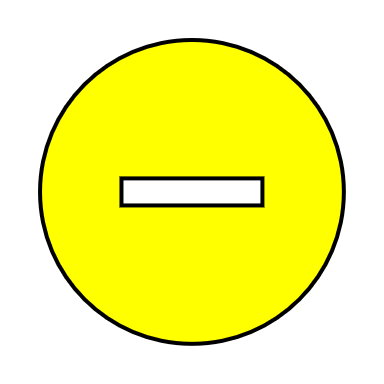 | 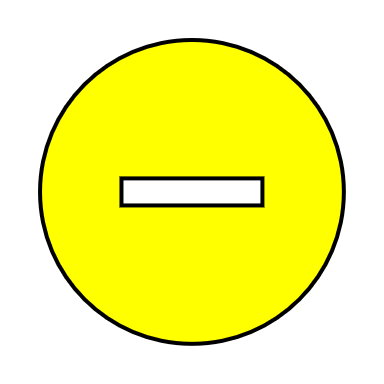 | 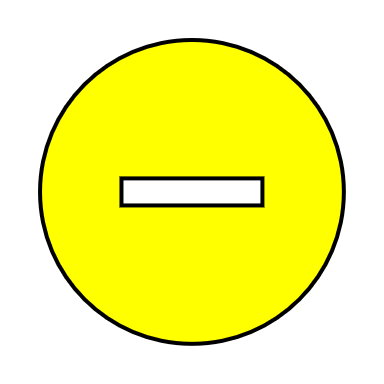 | 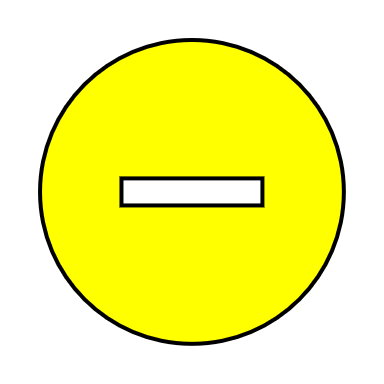 | 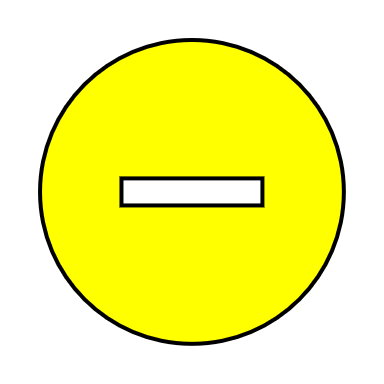 |
| Humeniuk et al. (2011) | 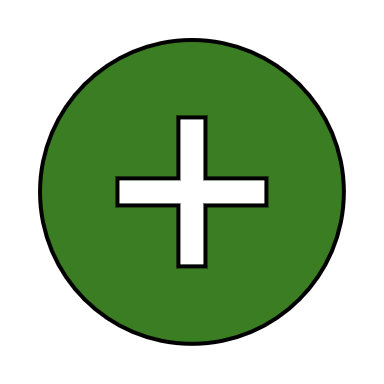 | 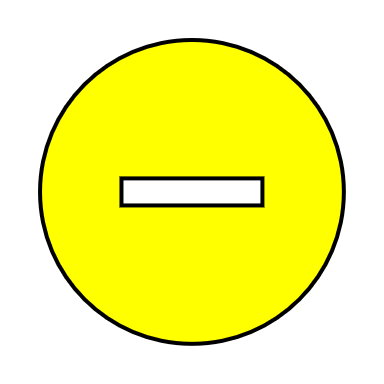 | 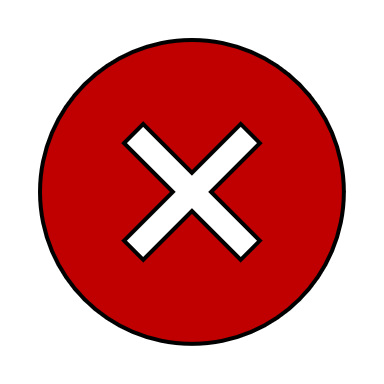 | 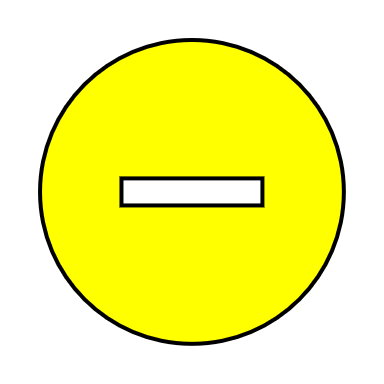 | 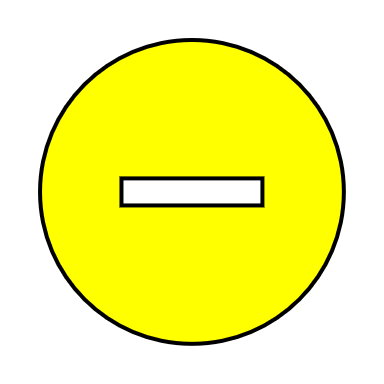 | 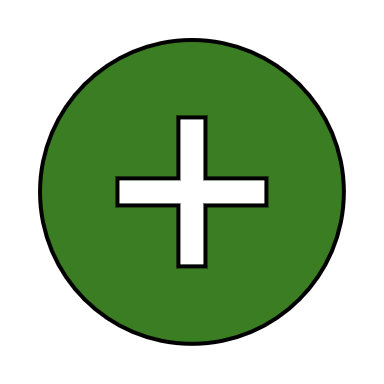 | 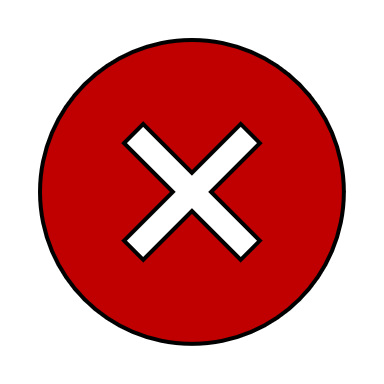 |
| Knight et al. (2019) | 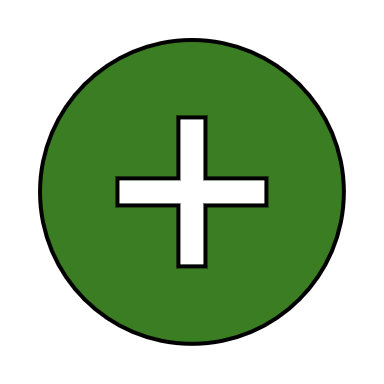 | 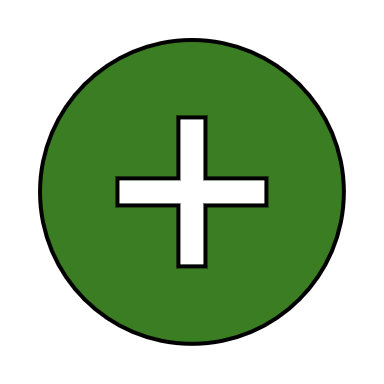 | 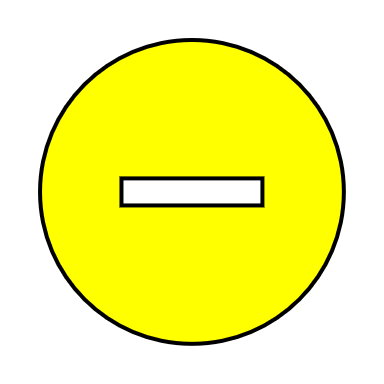 | 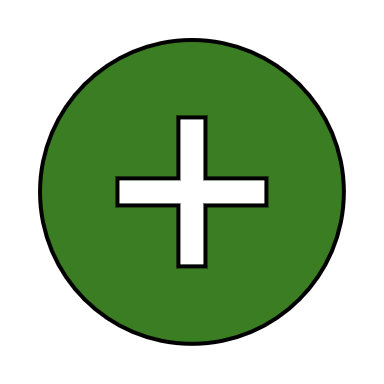 | 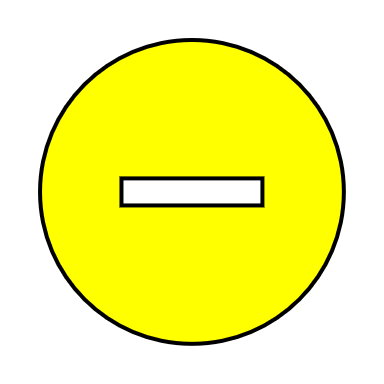 | 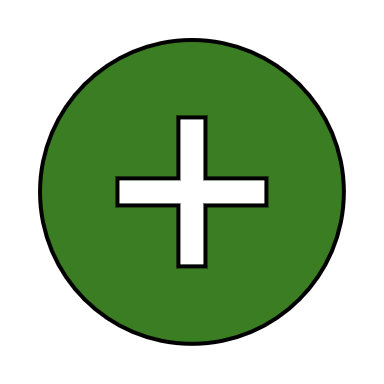 | 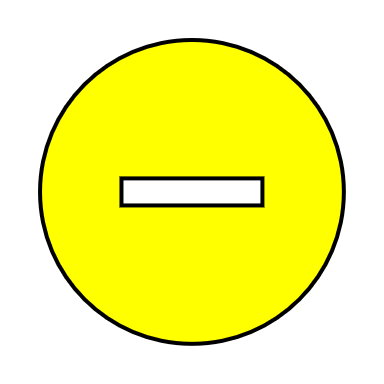 |
| Laporte et al. (2017) | 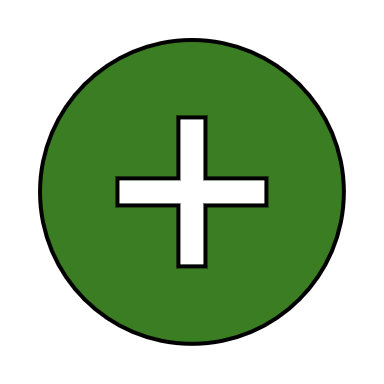 | 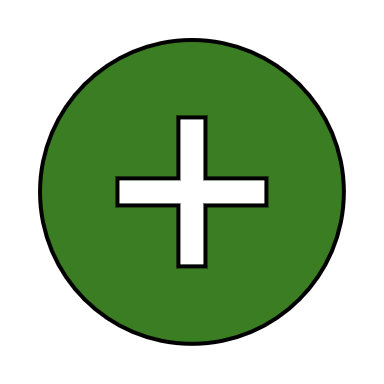 | 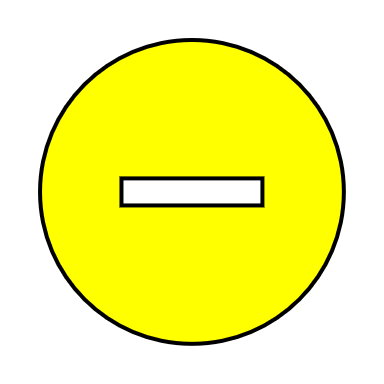 | 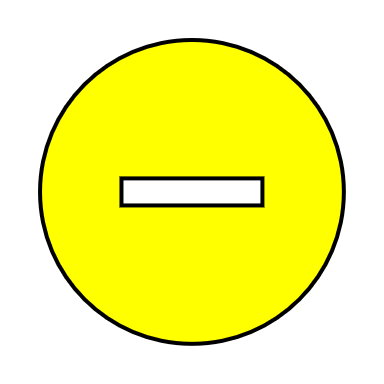 | 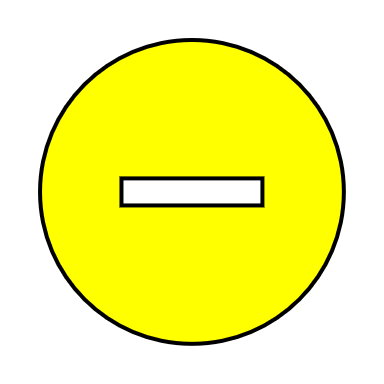 | 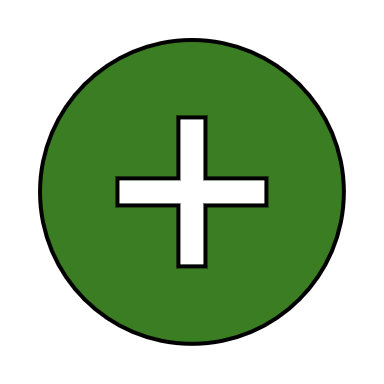 | 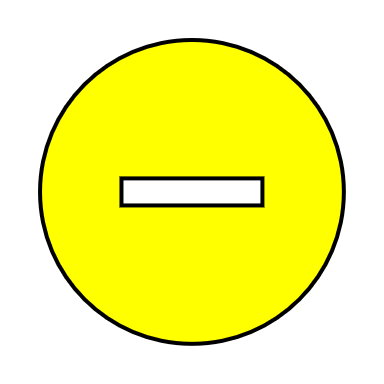 |
| Mason et al. (2015) | 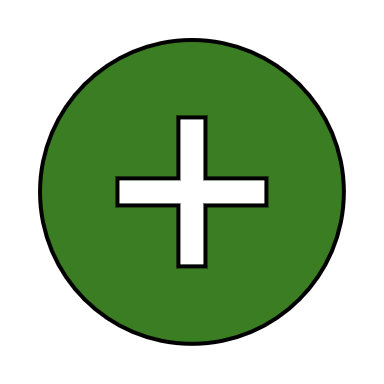 | 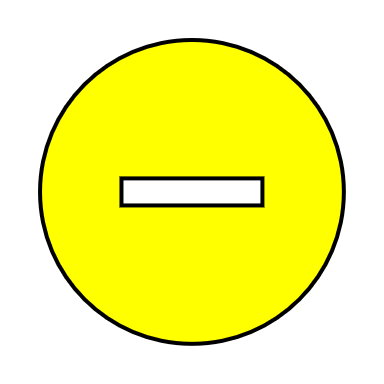 | 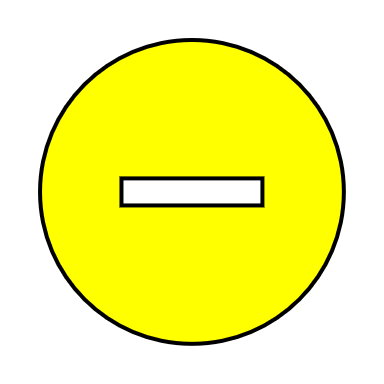 | 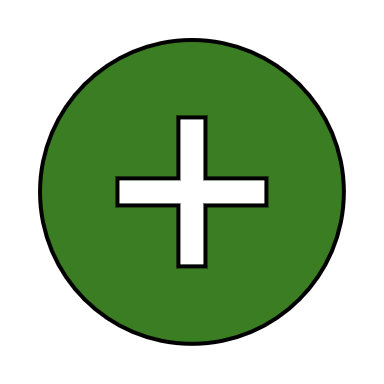 | 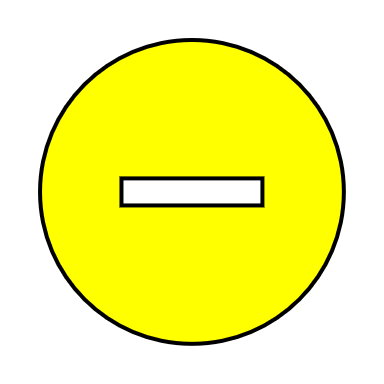 | 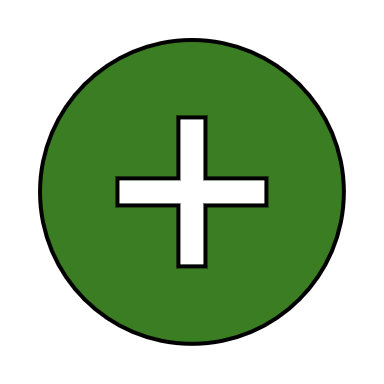 | 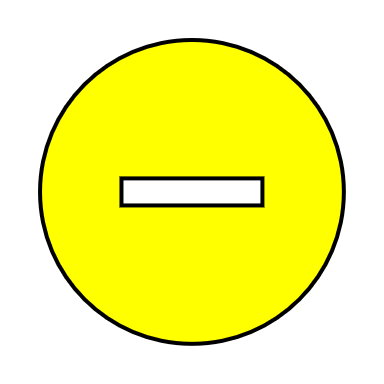 |
| Merchant et al. (2015) | 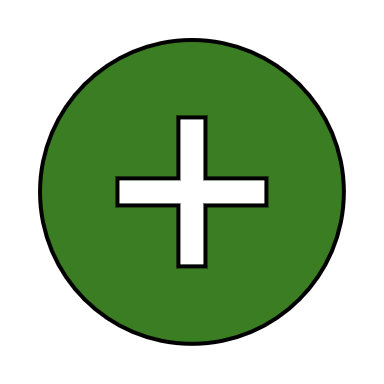 | 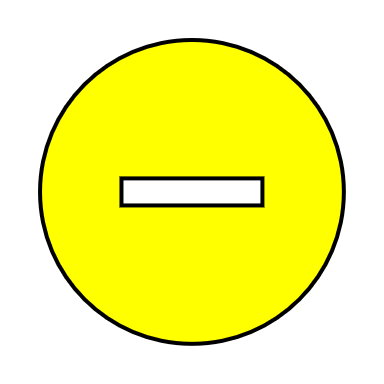 | 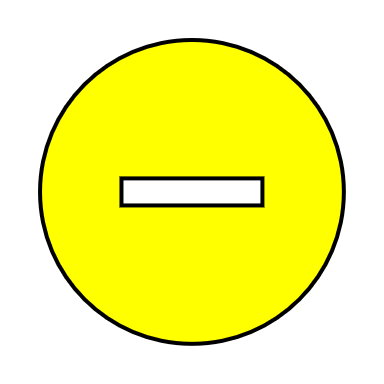 | 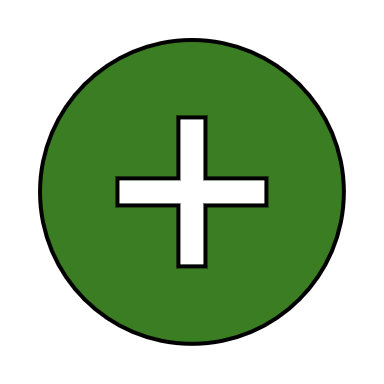 | 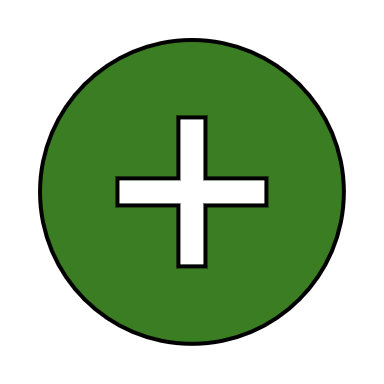 | 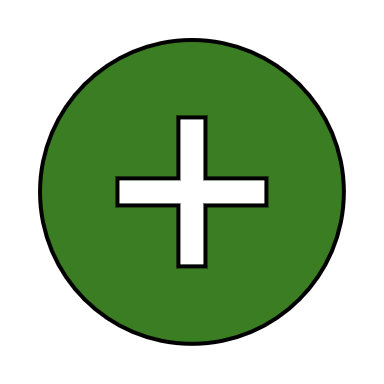 | 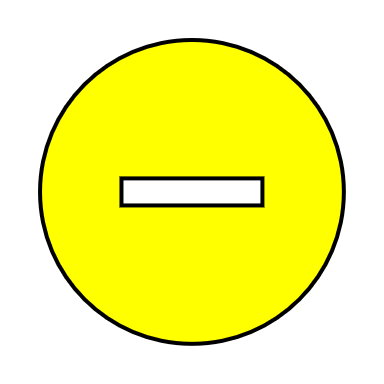 |
| Mertens et al. (2014) | 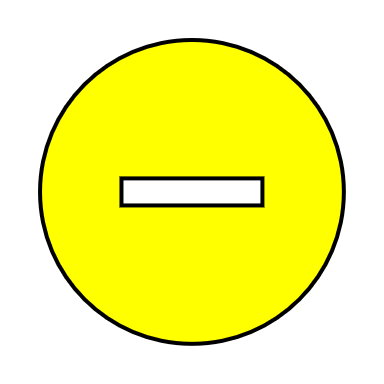 | 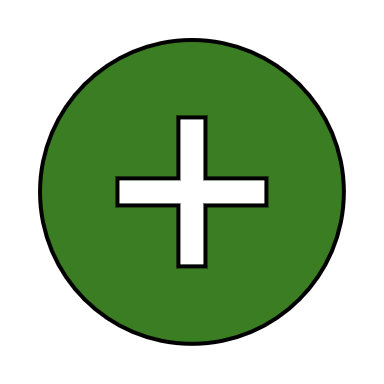 | 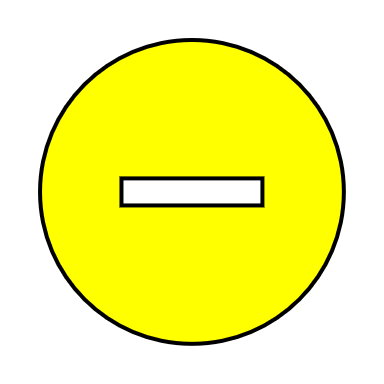 | 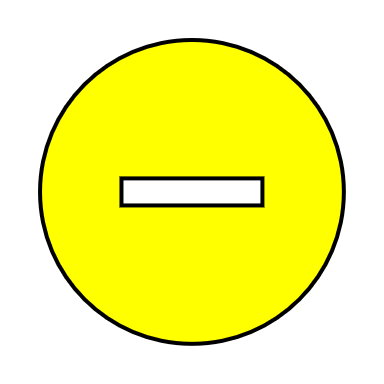 | 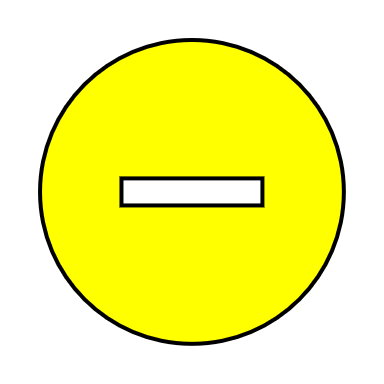 | 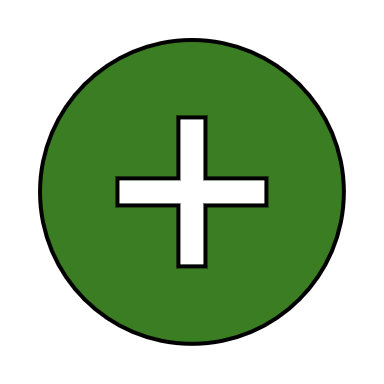 | 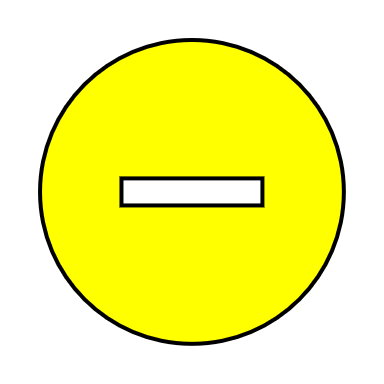 |
| Palfai et al. (2014) | 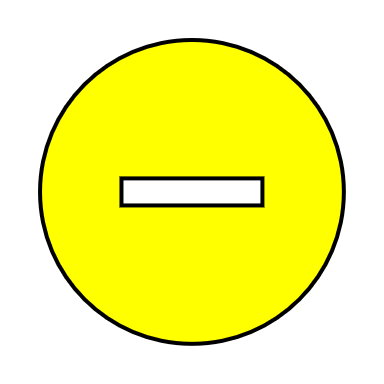 | 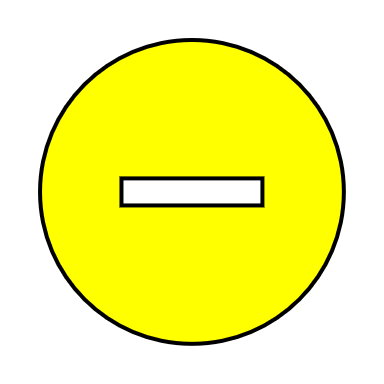 | 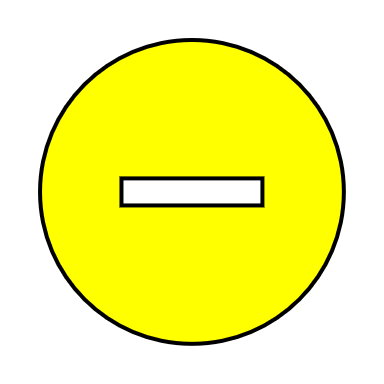 | 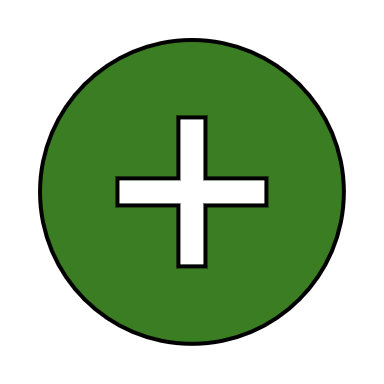 | 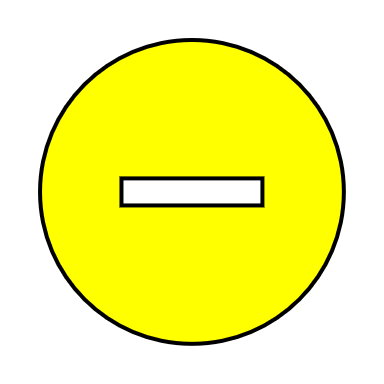 | 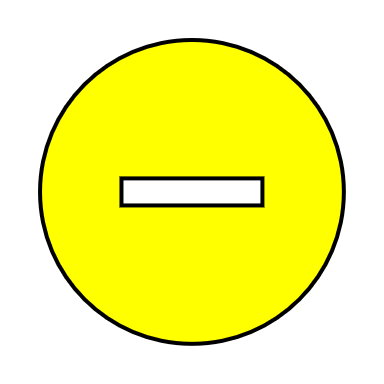 | 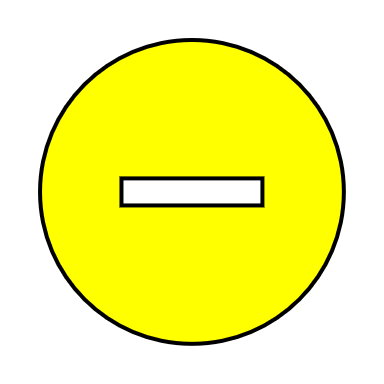 |
| Saitz et al. (2014) | 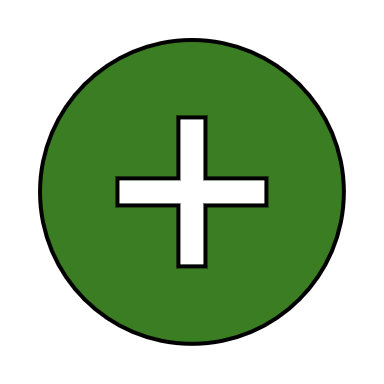 | 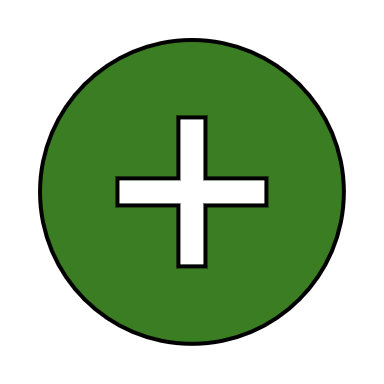 | 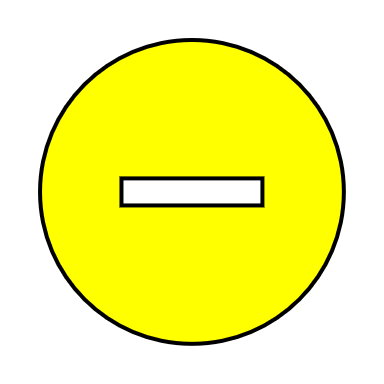 | 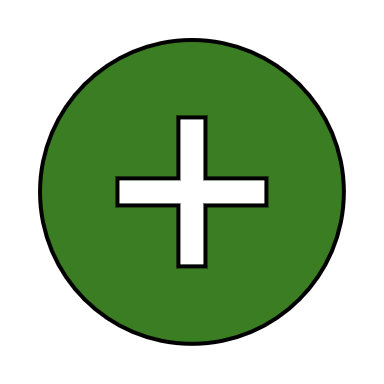 | 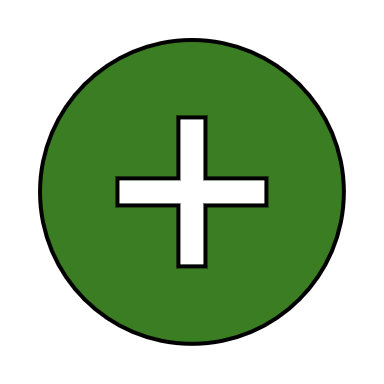 | 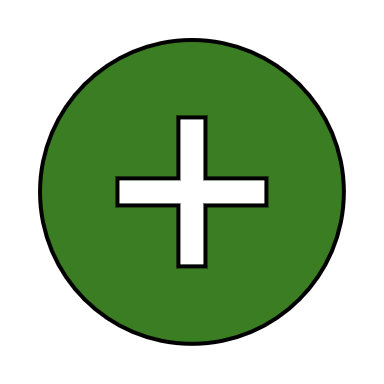 | 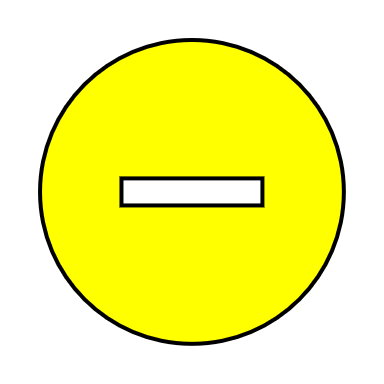 |
| Walsh et al. (2017) | 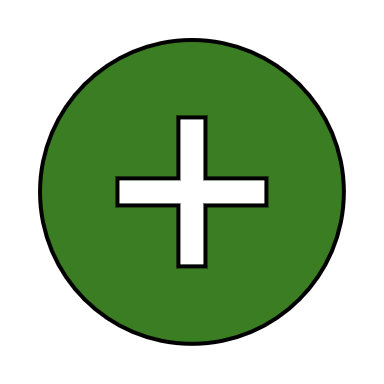 | 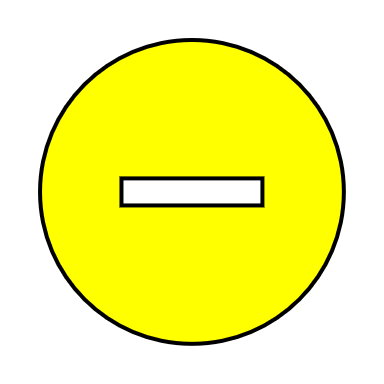 |  |  |  |  |  |
| Walton et al. (2013, 2014) |  |  |  |  |  |  |  |
| Woolard et al. (2013) |  |  |  |  |  |  |  |
| Domains |  |  |  |  | Judgement | | |
| D1: Random sequence generation  D2: Allocation concealment  D3: Blinding of Outcome assessment  D4: Incomplete outcome data  D5: Selective reporting  D6: Other bias | | |  |  |  | High | |
|  |  |  |  |  |  | Unclear | |
|  |  |  |  |  |  | Low | |
