# Supplementary material for: Brief Drug Interventions Delivered in General Medical Settings: a Systematic Review and Meta-analysis of Cannabis Use Outcomes
Source: Prev Sci. 2025 Jul 8;26(6):985–98. doi: 10.1007/s11121-025-01826-7 (PMC12394317; doi:10.1007/s11121-025-01826-7)
Supplement: Supplementary file 3 — Supplementary file3 (DOCX 1027 KB) [file 11121_2025_1826_MOESM3_ESM.docx]

**Supplemental Material S3: Forest Plots for Main Effect Analyses**

**Figure 1**

*Forest Plot of the Effects of Brief Drug Interventions of Short-Term Cannabis Consumption Level*


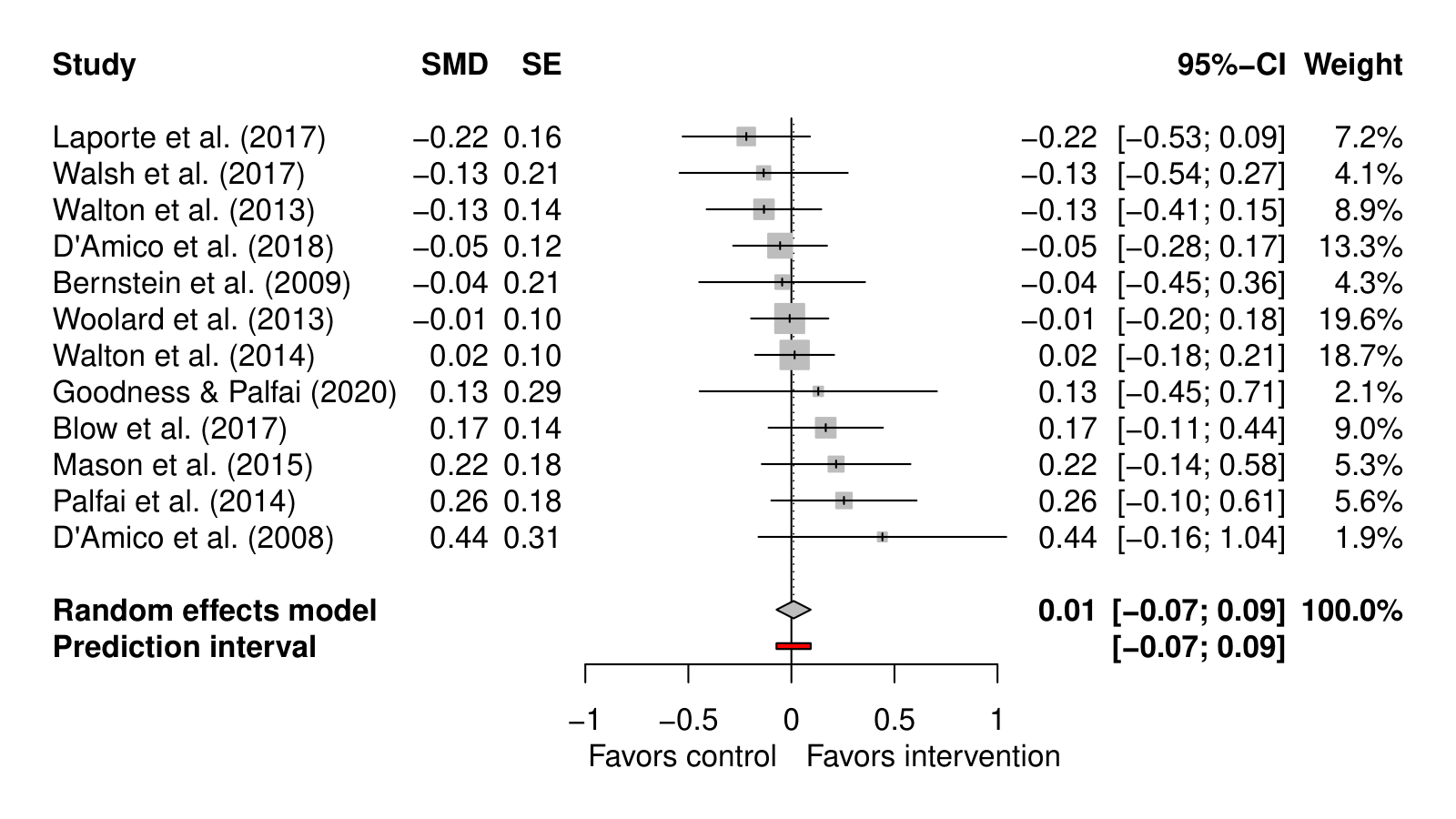


*Note*. SMD = Hedges’ *g* standardized mean difference; SE = standard error; CI = 95% confidence intervals.

**Figure 2**

*Forest Plot of the Effects of Brief Drug Interventions of Long-Term Cannabis Consumption Level*


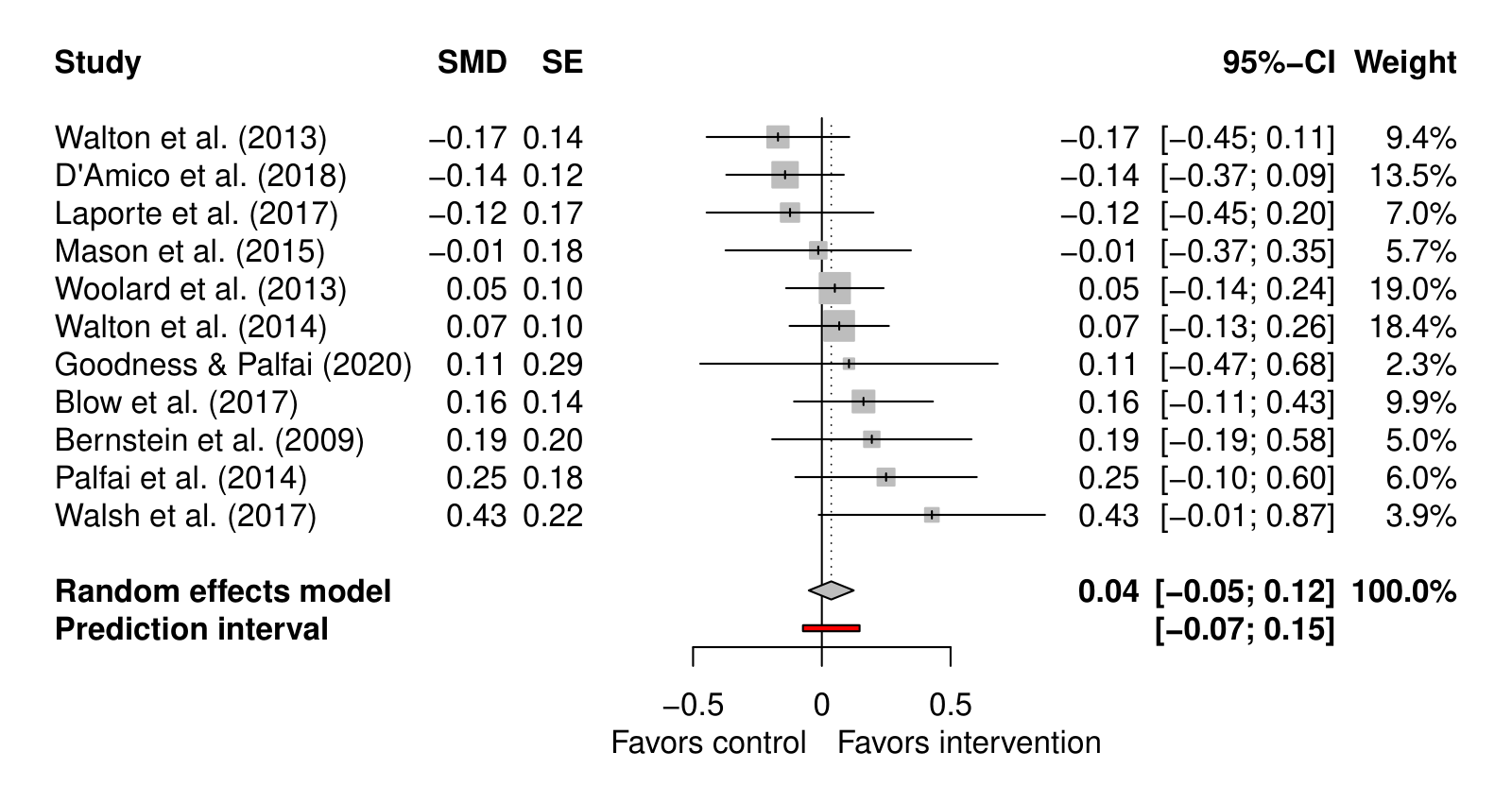


*Note*. SMD = Hedges’ *g* standardized mean difference; SE = standard error; CI = 95% confidence intervals.

**Figure 3**

*Forest Plot of the Effects of Brief Drug Interventions of Short-Term Cannabis Use*


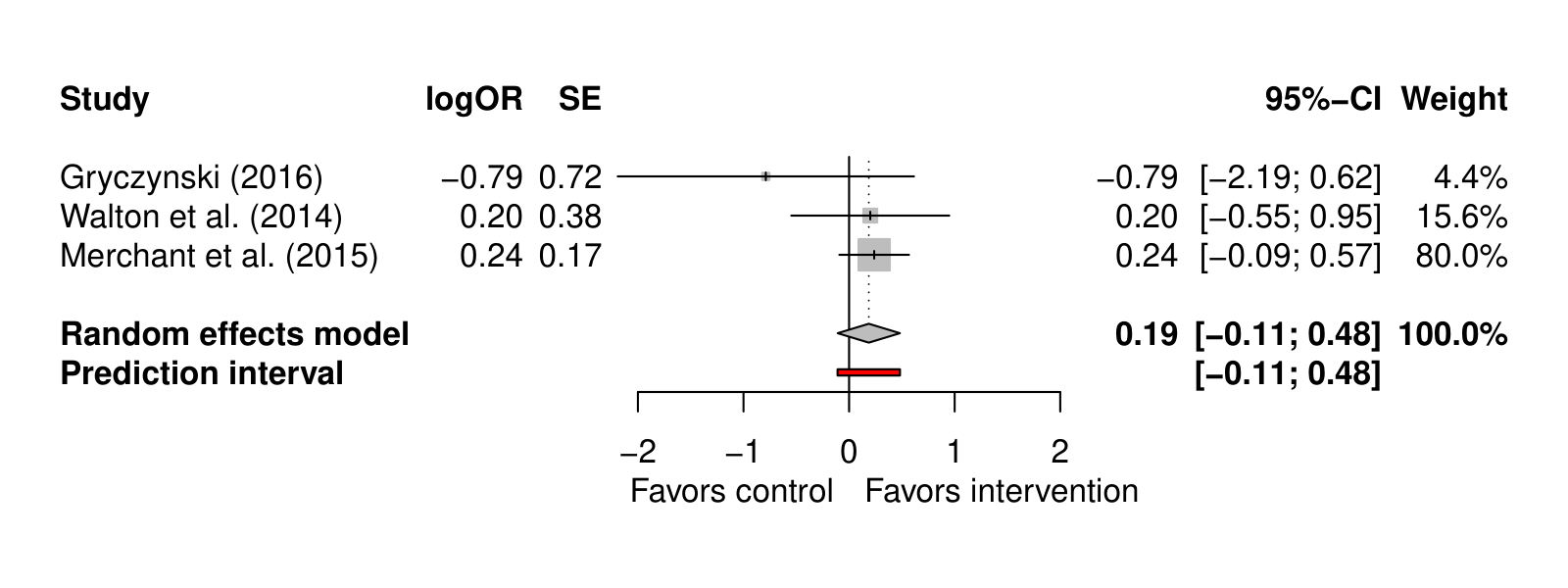


*Note*. logOR = log odds ratio; SE = standard error; CI = 95% confidence intervals.

**Figure 4**

*Forest Plot of the Effects of Brief Drug Interventions of Long-Term Cannabis Use*


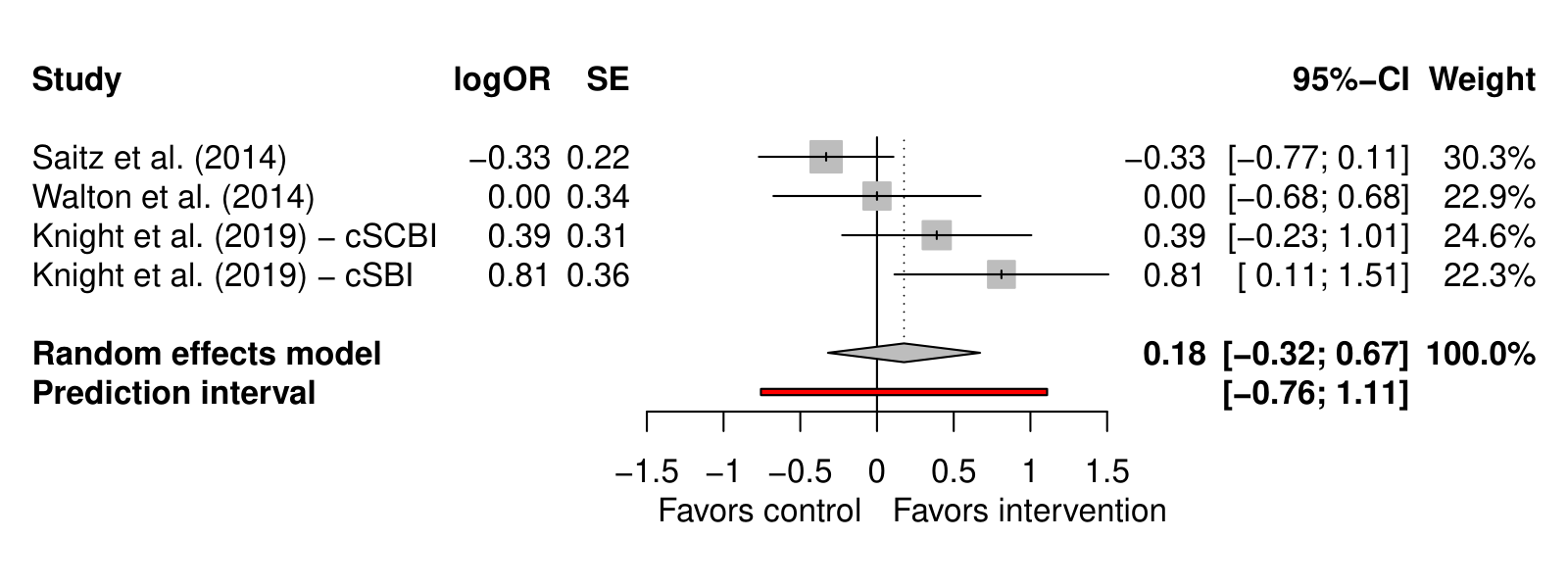


*Note*. logOR = log odds ratio; SE = standard error; CI = 95% confidence intervals.

**Figure 5**

*Forest Plot of the Effects of Brief Drug Interventions of Short-Term Cannabis Use Severity*


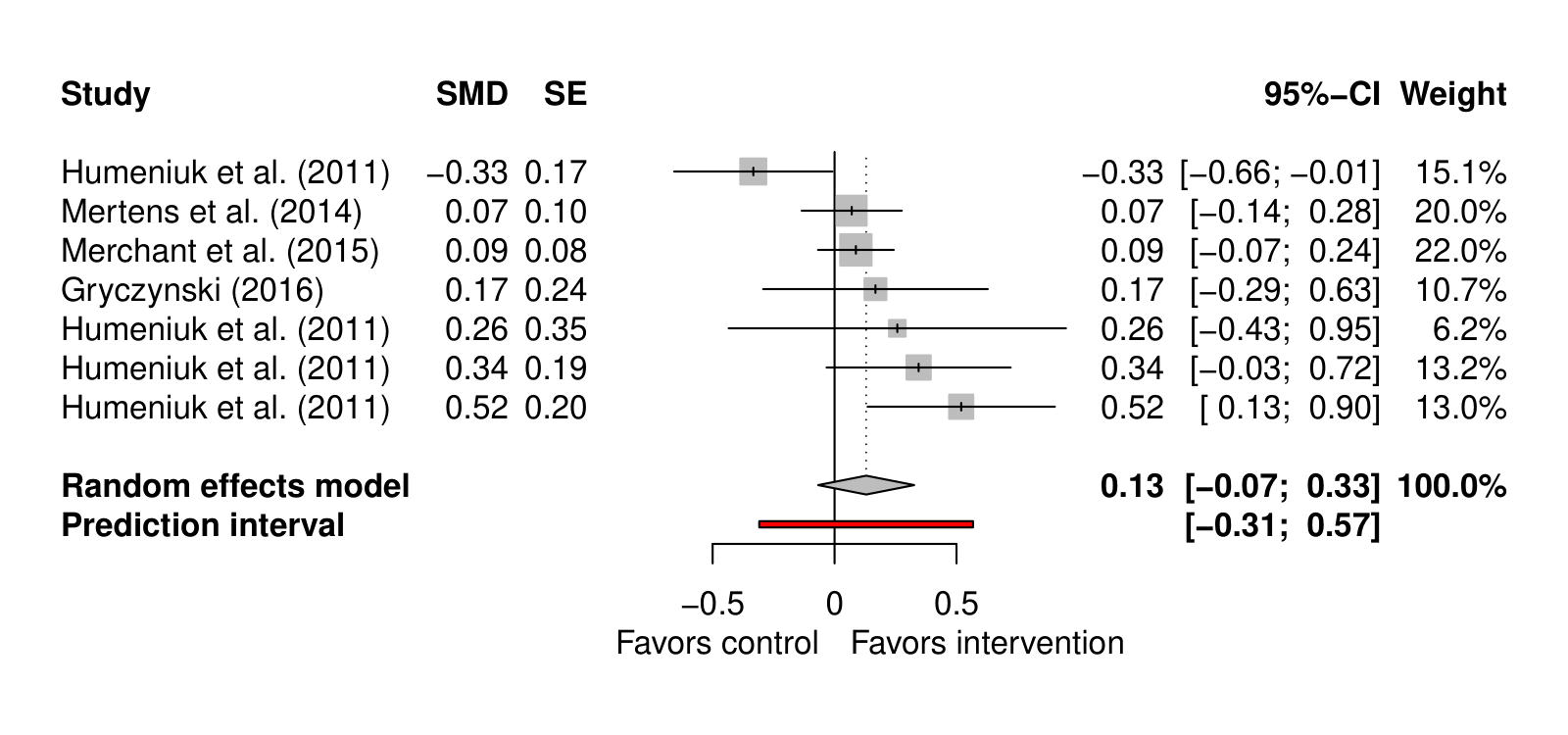


*Note*. SMD = Hedges’ *g* standardized mean difference; SE = standard error; CI = 95% confidence intervals.
