# Supplementary material for: Brief Drug Interventions Delivered in General Medical Settings: a Systematic Review and Meta-analysis of Cannabis Use Outcomes
Source: Prev Sci. 2025 Jul 8;26(6):985–98. doi: 10.1007/s11121-025-01826-7 (PMC12394317; doi:10.1007/s11121-025-01826-7)
Supplement: Supplementary file 4 — Supplementary file4 (DOCX 27 KB) [file 11121_2025_1826_MOESM4_ESM.docx]

**Supplemental Material S4: Main Effect Results From Sensitivity Tests**

There was no evidence of funnel plot asymmetry in the short-term (*z* = 1.08, *p* = .278) and long-term (*z* = 1.12, *p* = .261) consumption level outcomes, and trim and fill analyses did not yield different findings.

Despite between-study heterogeneity in long-term cannabis use consumption level not being statistically significant, the substantial increase in *I*^2^ values for the timepoint and intervention intensity sensitivity tests reflect greater variability those studies.

| **Table 4**  *Standardized Effect Sizes, 95% Confidence Intervals, and Heterogeneity Statistics by Outcome and Time Period for Sensitivity Tests* | | | | | | | | | |
| --- | --- | --- | --- | --- | --- | --- | --- | --- | --- |
|  |  | **0-5 Months** | | | | **6-12 Months** | | | |
| Sensitivity Test | Cannabis Outcome | ES [95% CI] | [95% PI] | *τ*^2^ | *I*^2^ | ES [95% CI] | [95% PI] | *τ*^2^ | *I*^2^ |
| Intervention intensity | Consumption level | 0.02 [-0.06, 0.11]_12_ | [-0.09, 0.14] | 0.00 | 5.90% | 0.06 [-0.05, 0.17]_11_ | [-0.18, 0.30] | 0.01 | 36.01% |
|  | Use | 0.19 [-0.11, 0.48]_3_ | [-0.11, 0.48] | 0.00 | 0.00% | 0.22 [-0.18, 0.63]_4_ | [-0.46, 0.91] | 0.08 | 46.48% |
|  |  |  |  |  |  |  |  |  |  |
| Timepoint | Consumption level | — | — | — | — | 0.03 [-0.09, 0.14]_11_ | [-0.23, 0.28] | 0.01 | 38.10% |
|  | Use | — | — | — | — | 0.24 [-0.24, 0.72]_4_ | [-0.67, 1.15] | 0.39 | 64.30% |
|  |  |  |  |  |  |  |  |  |  |
| Modality | Consumption level | 0.05 [-0.04. 0.13]_12_ | [-0.04, 0.13] | 0.00 | 0.00% | 0.08 [-0.13, 0.16] | [-0.04, 0.20] | 0.00 | 7.23% |
|  | Use | 0.23 [-0.07. 0.52]_3_ | [-0.07, 0.52] | 0.00 | 0.00% | 0.28 [-0.24, 0.79] | [-0.69, 1.25] | 0.18 | 64.80% |
| *Note*. Subscripts denote the number of independent effect sizes included for each outcome; consumption level and severity effect sizes are Hedges’ *g* and use effect sizes are logORs; ES = effect size; CI = 95% confidence intervals; PI = 95% prediction intervals. | | | | | | | | | |
