# Supplementary material for: Brief Drug Interventions Delivered in General Medical Settings: a Systematic Review and Meta-analysis of Cannabis Use Outcomes
Source: Prev Sci. 2025 Jul 8;26(6):985–98. doi: 10.1007/s11121-025-01826-7 (PMC12394317; doi:10.1007/s11121-025-01826-7)
Supplement: Supplementary file 5 — Supplementary file5 (DOCX 31 KB) [file 11121_2025_1826_MOESM5_ESM.docx]

**Supplemental Material S5: Moderation Results From Sensitivity Tests**

| **Table 1**  *Short-Term Subgroup Effect Sizes and 95% Confidence Intervals by Sensitivity Test* | | | | |
| --- | --- | --- | --- | --- |
|  | **Intensity** | | **Modality** | |
| Subgroup | Consumption Level | Use | Consumption Level | Use |
| Booster Session |  |  |  |  |
| No | 0.02 [-0.10, 0.13]_9_ | 0.03 [-0.07, 0.12]_1_ | 0.03 [-0.07, 0.12]_8_ | -0.11 [-5.93, 5.72]_1_ |
| Yes | 0.11 [-0.25, 0.48]_3_ | 0.15 [-0.06, 0.36]_2_ | 0.15 [-0.06, 0.36]_4_ | 0.24 [-5.27, 5.75]_2_ |
|  |  |  |  |  |
| Setting |  |  |  |  |
| Community health center | -0.02 [-0.16, 0.12]_6_ | 0.04 [-0.09, 0.17]_2_ | 0.04 [-0.09, 0.17]_6_ | -0.11 [-5.93, 5.71]_2_ |
| Emergency department | 0.05 [-0.13, 0.22]_4_ | 0.02 [-0.13, 0.18]_1_ | 0.02 [-0.13, 0.18]_4_ | 0.24 [-5.27, 5.75]_1_ |
| Hospital-based primary care | ― | ― | ― | ― |
| Multiple settings | ― | ― | ― | ― |
| University health center | 0.22 [-0.15, 0.59]_2_ | ― | 0.22 [-0.13, 0.57]_2_ | ― |
|  |  |  |  |  |
| Target |  |  |  |  |
| Other drugs/mixed substances | 0.10 [-0.07, 0.28]_6_ | 0.07 [-0.09, 0.24]_2_ | 0.07 [-0.09, 0.24]_6_ | -0.04 [-5.83, 5.75]_2_ |
| Cannabis | -0.02 [-0.14, 0.10]_6_ | 0.03 [-0.08, 0.15]_1_ | 0.03 [-0.08, 0.15]_6_ | 0.20 [-7.85, 8.26]_1_ |
|  |  |  |  |  |
| Population |  |  |  |  |
| Adolescents only | 0.01 [-0.14, 0.16]_5_ | 0.08 [-0.04, 0.20]_1_ | 0.08 [-0.04, 0.20]_5_ | 0.20 [-7.85, 8.26]_1_ |
| Adolescents and young adults | -0.15 [-0.45, 0.14]_2_ | ― | -0.15 [-0.41, 0.10]_2_ | ― |
| Mixed and adults only | 0.06 [-0.13, 0.25]_3_ | 0.03 [-0.12, 0.18]_2_ | 0.03 [-0.12, 0.18]_3_ | -0.04 [-5.83, 5.75]_2_ |
| University students | 0.22 [-0.14, 0.58]_2_ | ― | 0.22 [-0.09, 0.53]_2_ | ― |
| *Note*. Subscripts denote the number of independent effect sizes in each subgroup; consumption level and severity effect sizes are Hedges’ *g* and use effect sizes are logORs. | | | | |

| **Table 2**  *Long-Term Subgroup Effect Sizes and 95% Confidence Intervals by Sensitivity Test* | | | | | | |
| --- | --- | --- | --- | --- | --- | --- |
|  | **Intensity** | | **Timepoint** | | **Modality** | |
| Subgroup | Consumption Level | Use | Consumption Level | Use | Consumption Level | Use |
| Booster Session |  |  |  |  |  |  |
| No | 0.05 [-0.09, 0.19]_9_ | ― | -0.02 [-0.16, 0.12]_8_ | 0.46 [-0.20, 1.13]_1_ | 0.05 [-0.06, 0.16]_8_ | 0.53 [-0.03, 1.10]_1_ |
| Yes | 0.16 [-0.26, 0.58]_2_ | ― | 0.19 [-0.10, 0.45]_3_ | -0.33 [-1.13, 0.47]_3_ | 0.19 [-0.04, 0.43]_3_ | -0.33 [-0.97, 0.31]_3_ |
|  |  |  |  |  |  |  |
| Setting |  |  |  |  |  |  |
| Community health center | -0.06 [-0.18, 0.06]_5_ | 0.00 [-3.89, 3.89]_1_ | -0.11 [-0.24, 0.02]_5_ | 0.28 [-3.12, 3.69]_1_ | -0.00 [-0.13, 0.12]_5_ | 0.44 [-3.77, 4.66]_1_ |
| Emergency department | 0.19 [0.04, 0.35]*_4_ | ― | 0.15 [0.00, 0.30]*_4_ | ― | 0.16 [0.01, 0.31]*_4_ | ― |
| Hospital-based primary care | ― | -0.11 [-2.61, 2.38]_1_ | ― | -0.33 [-2.86, 2.20]_1_ | ― | -0.33 [-2.86, 2.20]_1_ |
| Multiple settings | ― | 0.57 [-2.09, 3.23]_2_ | ― | 0.57 [-2.09, 3.23]_2_ | ― | 0.57 [-2.09, 3.23]_2_ |
| University health center | 0.21 [-0.08, 0.50]_2_ | ― | 0.21 [-0.11, 0.53]_2_ | ― | 0.21 [-0.11, 0.52]_2_ | ― |
|  |  |  |  |  |  |  |
| Target |  |  |  |  |  |  |
| Other drugs/mixed substances | 0.11 [-0.11, 0.33]_5_ | 0.31 [-0.85, 1.47]_3_ | 0.06 [-0.18, 0.30]_5_ | 0.25 [-1.21, 1.70]_3_ | 0.06 [-0.12, 0.25]_5_ | 0.25 [-1.21, 1.70]_3_ |
| Cannabis | 0.03 [-0.11, 0.19]_6_ | 0.00 [-2.15, 2.15]_1_ | 0.01 [-0.17, 0.19]_6_ | 0.28 [-2.24, 2.80]_1_ | 0.09 [-0.05, 0.22]_6_ | 0.44 [-2.25, 3.13]_1_ |
|  |  |  |  |  |  |  |
| Population |  |  |  |  |  |  |
| Adolescents only | -0.06 [-0.21, 0.10]_4_ | 0.39 [-0.58, 1.37]_3_ | -0.10 [-0.27, 0.08]_4_ | 0.46 [-0.20, 1.13]_3_ | 0.01 [-0.15, 0.18]_4_ | 0.54 [-0.03, 1.10]_3_ |
| Adolescents and young adults | 0.01 [-0.28, 0.30]_2_ | ― | -0.04 [-0.36, 0.29]_2_ | ― | 0.01 [-0.29, 0.31]_2_ | ― |
| Mixed and adults only | 0.20 [-0.00, 0.41]_3_ | 0.11 [-1.38, 1.15]_1_ | 0.16 [-0.05, 0.37]_3_ | -0.33 [-1.13, 0.47]_1_ | 0.16 [-0.03, 0.35]_3_ | -0.33 [-0.97, 0.31]_1_ |
| University students | 0.21 [-0.14, 0.55]_2_ | ― | 0.21 [-0.18, 0.59]_2_ | ― | 0.21 [-0.15, 0.56]_2_ | ― |
| *Note*. Subscripts denote the number of independent effect sizes in each subgroup; consumption level and severity effect sizes are Hedges’ *g* and use effect sizes are logORs. | | | | | | |
